# Symbiotic microbiota may reflect host adaptation by resident to invasive ant species

**DOI:** 10.1371/journal.ppat.1007942

**Published:** 2019-07-19

**Authors:** Daifeng Cheng, Siqi Chen, Yuquan Huang, Naomi E. Pierce, Markus Riegler, Fan Yang, Ling Zeng, Yongyue Lu, Guangwen Liang, Yijuan Xu

**Affiliations:** 1 Department of Entomology, South China Agricultural University, Guangzhou, China; 2 Department of Organismic and Evolutionary Biology, Harvard University, Cambridge MA, United States of America; 3 Hawkesbury Institute for the Environment, Western Sydney University, Penrith, NSW, Australia; Stanford University, UNITED STATES

## Abstract

Exotic invasive species can influence the behavior and ecology of native and resident species, but these changes are often overlooked. Here we hypothesize that the ghost ant, *Tapinoma melanocephalum*, living in areas that have been invaded by the red imported fire ant, *Solenopsis invicta*, displays behavioral differences to interspecific competition that are reflected in both its trophic position and symbiotic microbiota. We demonstrate that *T*. *melanocephalum* workers from *S*. *invicta* invaded areas are less aggressive towards workers of *S*. *invicta* than those inhabiting non-invaded areas. Nitrogen isotope analyses reveal that colonies of *T*. *melanocephalum* have protein-rich diets in *S*. *invicta* invaded areas compared with the carbohydrate-rich diets of colonies living in non-invaded areas. Analysis of microbiota isolated from gut tissue shows that *T*. *melanocephalum* workers from *S*. *invicta* invaded areas also have different bacterial communities, including a higher abundance of *Wolbachia* that may play a role in vitamin B provisioning. In contrast, the microbiota of workers of *T*. *melanocephalum* from *S*. *invicta*-free areas are dominated by bacteria from the orders Bacillales, Lactobacillales and Enterobacteriales that may be involved in sugar metabolism. We further demonstrate experimentally that the composition and structure of the bacterial symbiont communities as well as the prevalence of vitamin B in *T*. *melanocephalum* workers from *S*. *invicta* invaded and non-invaded areas can be altered if *T*. *melanocephalum* workers are supplied with either protein-rich or carbohydrate-rich food. Our results support the hypothesis that bacterial symbiont communities can help hosts by buffering behavioral changes caused by interspecies competition as a consequence of biological invasions.

## Author summary

Insects display a wide range of dependence on symbiotic bacteria for basic functions. Responses by resident species to selective pressures imposed by invasive species, as well as specific underlying mechanisms that give rise to these responses are still poorly understood. Here we investigate the role of the symbiotic bacteria of the ghost ant, *Tapinoma melanocephalum*, to changes in host behavior associated with interspecies competition in areas invaded by fire ants, *Solenopsis invicta*. We show that *Wolbachia* is significantly enriched in workers of *T*. *melanocephalum* from *S*. *invicta* infested areas, and that these bacteria also increase in abundance in colonies that have been supplied with protein-rich food. Our results suggest that bacterial symbiont communities can play an important role in enabling ants to tolerate changes in behavior and diet as a result of biological invasions.

## Introduction

Rapid development of global trade and travel have created conditions for long-distance migration and concomitantly increased the threat of biological invasion by exotic species [1]. Invasive species can affect the distribution, abundance and reproduction of native taxa [2,3] and disturb the structure and function of ecosystems [4]. Invasive species can also cause severe economic losses in agriculture, forestry and fishery, and potentially threaten the health of humans [5,6].

Invasive ants are among the greatest threats to ecosystems; dozens of species have invaded islands and continents around the world [7]. In particular, the red imported fire ant, *Solenopsis invicta* Buren, relies on behavioral and numerical dominance to displace endemic, native and other locally occurring taxa, including previously introduced species (hereafter referred to collectively as “resident” species) across its introduced range [8]. Once established, *S*. *invicta* acts as an omnivore and ecosystem engineer, with dramatic effects on ecologically similar resident ants. In the United States, the congeneric species *S*. *xyloni* McCook and *S*. *geminata* Fabricius appear to be particularly sensitive to displacement by *S*. *invicta* [9]. In central Texas, *S*. *invicta* has also been reported to destroy and eradicate colonies of the harvester ant, *Pogonomyrmex barbatus* Smith [10].

With their superior competitive abilities, invasive species impose strong selection on resident taxa, some of which have been shown to adapt to these pressures in different ways [11]. To persist in invaded areas, common adaptive behavioral responses by resident species include altered anti-predator defenses [12,13] and changes in the spectrum of used resources [14] and habitats [15]. Recognizing these interactions is critical to understanding the long-term impacts of biological invasions. However, responses by resident species to selective pressures imposed by invasive species, as well as specific underlying mechanisms that give rise to these responses are still poorly understood.

Symbiotic bacteria can be essential for the growth and survival of their hosts [16–18]. They can play an integral role in the breakdown of food, recycling and provision of energy, production of vitamins, and even shape innate immunity [19–22]. Microbial symbionts have been shown to have broad effects on the health and behavior in humans and other mammals [23]. In the context of biological invasions, they have largely been studied to identify or assess their effects in enhancing the invasion process of introduced species [24–27]. A better understanding of the composition and function of bacterial symbionts, however, might also reveal potential mechanisms for behavioral change of resident host species to exotic invasives, since changes in the bacterial symbionts have been shown to correspond to changes in food resources in both vertebrates and invertebrates [28–33]. Furthermore, characterizing the bacterial symbionts of resident species could provide important clues about how to manage invasive species.

*Tapinoma melanocephalum* (Fabricius) (Hymenoptera: Formicidae), the ghost ant, is a cosmopolitan ant species that is common in southern China. It was first recorded in China in 1921 and is likely to have originated in the Indo-Pacific region [34]. It has successfully invaded both human-disturbed and undisturbed natural habitats of tropical and subtropical regions of the world [34–36]. *Tapinoma melanocephalum* colonies are typically polygynous, unicolonial and resilient to disturbance [37], and therefore display key features of successful invasive ants [38–40]. Although interactions between invasive and resident ants are well documented [41,42], examples of interactions between two different invasive ant species are rare. In a previous field investigation, we found that *T*. *melanocephalum* often persists in areas invaded by the fire ant *S*. *invicta*, first recorded in mainland China in 2004 [43]. Due to their aggressiveness and capacity to reach high population densities, most available food resources can be used by workers of *S*. *invicta* [44,45]. In the field, workers of *S*. *invicta* can outcompete those of *T*. *melanocephalum* in the use of available honeydew and thus may coerce *T*. *melanocephalum* into utilizing a different ecological niche [44,45]. Here, we examined three main hypotheses: (1) Workers of *T*. *melanocephalum* may exhibit less aggression in response to invasions by *S*. *invicta*. (2) Colonies of *T*. *melanocephalum* living in *S*. *invicta* invaded areas have a different diet than those inhabiting non-invaded areas, presumably due to competition for resources. (3) The bacterial community found in workers of *T*. *melanocephalum* in *S*. *invicta* invaded areas differs from that found in workers in non-invaded areas, and these differences are associated with their difference in diet.

To address these hypotheses, we investigated several possible mechanisms involved in the behavioral differences of *T*. *melanocephalum* following invasions by *S*. *invicta*. Stable isotope analysis can reveal differences in feeding activities [46], and previous research has indicated that δN is typically correlated with the trophic level and nutritional state of an organism [47]. We examined stable isotope composition and symbiotic bacterial communities of *T*. *melanocephalum* workers from *S*. *invicta* invaded and non-invaded sites, and measured responses of colonies to different diets in an attempt to simulate the field conditions. The laboratory simulation provided additional support for the field observations as we were not allowed to introduce *S*. *invicta* into non-invaded sites. Thus we did not directly assess the situation before and after invasion by *S*. *invicta* at individual sites, but assessed effects by comparing invaded and non-invaded sites in combination with laboratory experiments. The results provide insights into mechanisms of host responses to interspecific competition by two co-occurring ant species with invasive traits.

## Results

### Species composition of resident ant communities are significantly different between *S*. *invicta* invaded and non-invaded areas

**Fig 1:**
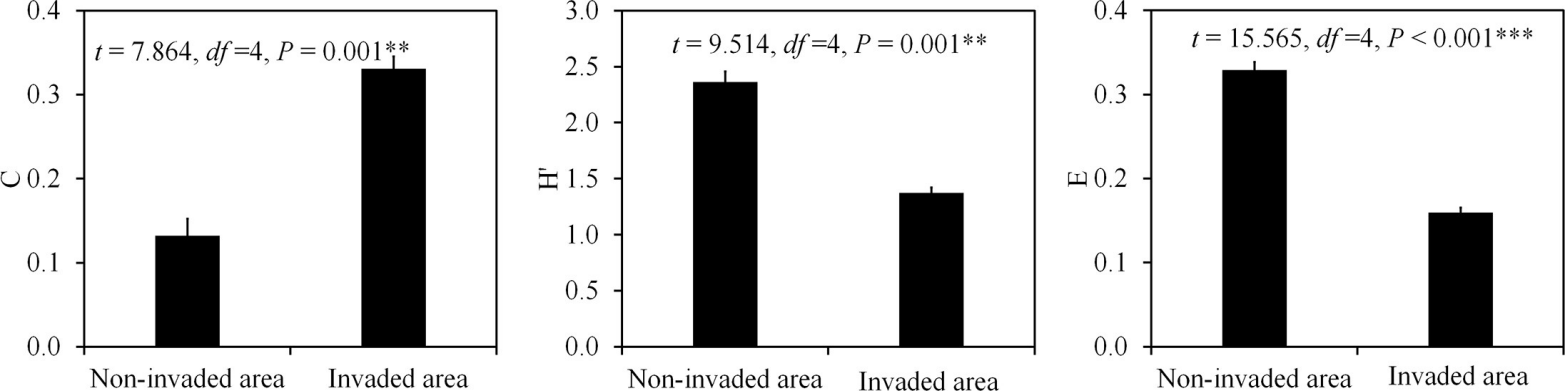
Effect of *S*. *invicta* invasion on biodiversity of resident ant species (Independent sample t test, mean±SE, *n* = 3 biological replicates). Asterisks indicate significant differences (***P < 0.001). C: Simpson dominance index; H’: Shannon-Wiener index; E: the Pielou evenness index.

By trapping and baiting, 3,947 workers belonging to 22 ant species were collected in the *S*. *invicta* non-invaded areas, while 8,005 *S*. *invicta* workers and 8,412 workers belonging to 14 other ant species were collected in the *S*. *invicta* invaded areas. We found that the numbers of workers of several ant species, especially *T*. *melanocephalum* in the *S*. *invicta* invaded areas, were significantly higher (626 *T*. *melanocephalum* in the non-invaded areas versus 4,034 *T*. *melanocephalum* in the invaded areas) (S1 Table). Compared with the non-invaded areas, the Simpson dominance index (*C*) was significantly higher in the invaded areas, while the Shannon-Wiener index (*H’*) and Pielou evenness index (*E*) were significantly higher in the non-invaded areas (Fig 1). These results indicate that species composition of resident ant communities are significantly different between *S*. *invicta* invaded and non-invaded areas, and *S*. *invicta* invasion may be one reason for the differences.

### *Tapinoma melanocephalum* workers exhibit less aggression in areas inhabited by *S*. *invicta*

**Fig 2:**
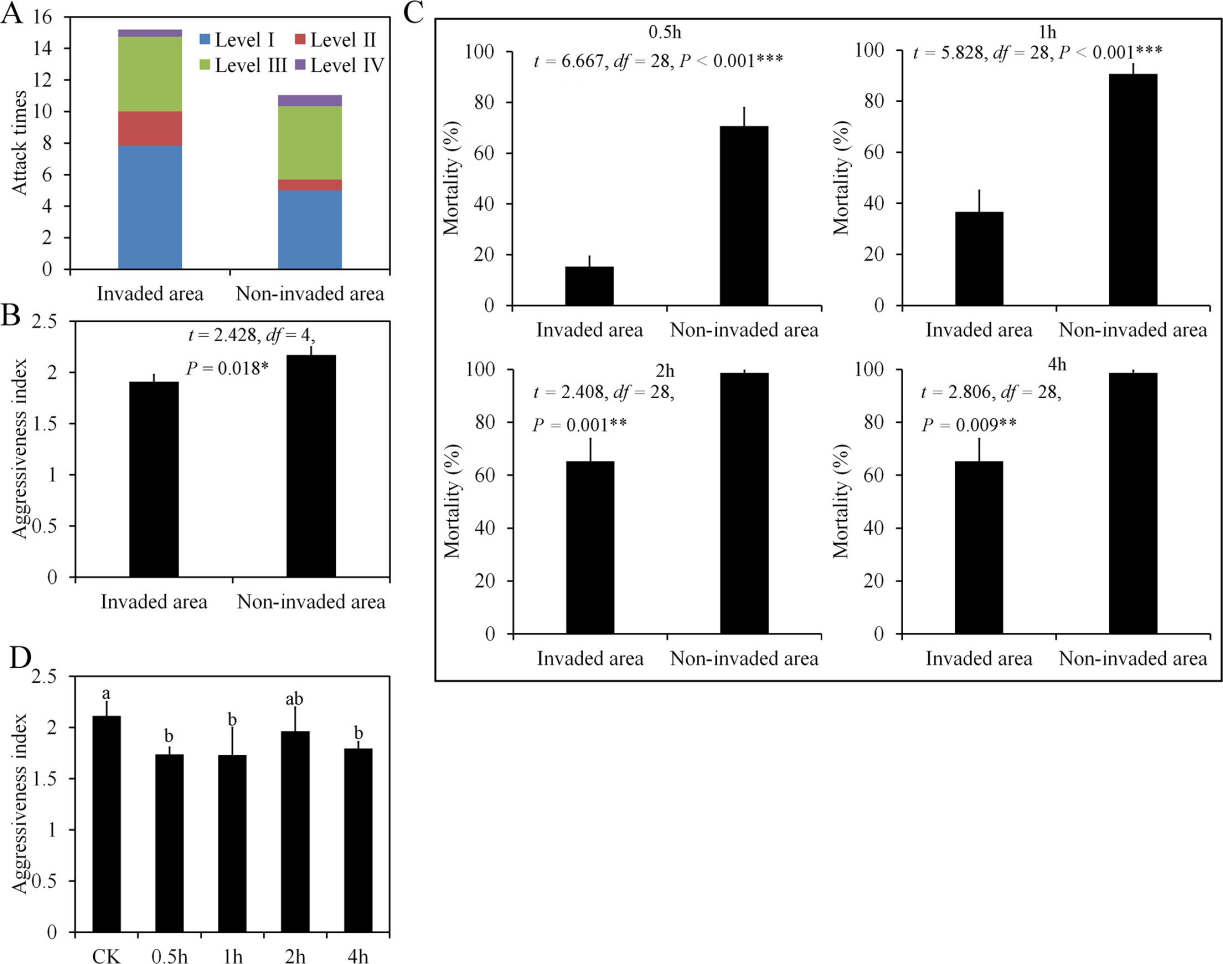
Individual and group behavior of workers of *T*. *melanocephalum* from either invaded or non-invaded areas when attacked by *S*. *invicta* workers. (A) Attack level (No significant difference of attack levels was found between invaded and non-invaded areas except Level II; Level III: t = 0.044, df = 2, P = 0.178; Level II: t = 4.612, df = 2, P = 0.044; Level III: t = 0.143, df = 2, P = 0.899; Level IV: t = -1.0, df = 2, P = 0.423) and (B) aggressiveness index (Independent sample t test, mean ± SE, *n* = 3 biological replicates) between *S*. *invicta* and *T*. *melanocephalum* from *S*. *invicta* invaded and non-invaded areas. (C) Group mortality (Independent sample t test, mean ± SE, *n* = 15 biological replicates) of *T*. *melanocephalum* under attack by *S*. *invicta* workers in *S*. *invicta* invaded and non-invaded areas. (D) Aggressiveness index (One-Way ANOVA, mean ± SE, bars with same letters indicate no significant difference (*P* > 0.05)) for *T*. *melanocephalum* from the non-invaded area at different times following contact with *S*. *invicta*. CK: *T*. *melanocephalum* without previous contact with *S*. *invicta*; Asterisks indicate significant differences (*P < 0.05, **P < 0.01, ***P < 0.001).

A comparison of the aggressiveness index based on encounters between *T*. *melanocephalum* workers from invaded and non-invaded areas with *S*. *invicta* shows that most encounters involved lower levels of attack (level I and level II), accounting for 66% and 52% of the total scores in invaded and non-invaded areas, respectively (Fig 2A). Based on the aggressiveness index, *T*. *melanocephalum* workers inhabiting *S*. *invicta* invaded areas displayed lower levels of antagonism compared with those inhabiting non-invaded areas (Fig 2B).

In the group aggression experiment, *T*. *melanocephalum* workers from the *S*. *invicta* non-invaded areas showed higher rates of mortality than those from the *S*. *invicta* invaded areas at 0.5h, 1h, 2h and 4h (Fig 2C). Moreover, after first contact with workers of *S*. *invicta*, the *T*. *melanocephalum* workers from the non-invaded areas had significant lower attack indexes at 0.5 h, 1 h and 4 h than those without contact experience (*F*_4, 70_ = 4.411, *P* = 0.003) (Fig 2D). These results support the hypothesis that *T*. *melanocephalum* workers living in habitats invaded by *S*. *invicta* display submissive behaviors in order to avoid attack by fire ants.

### Stable isotope analysis reveals differences in the feeding behavior of colonies of *T*. *melanocephalum* that may result from interspecific competition with invading colonies of *S*. *invicta*

**Fig 3:**
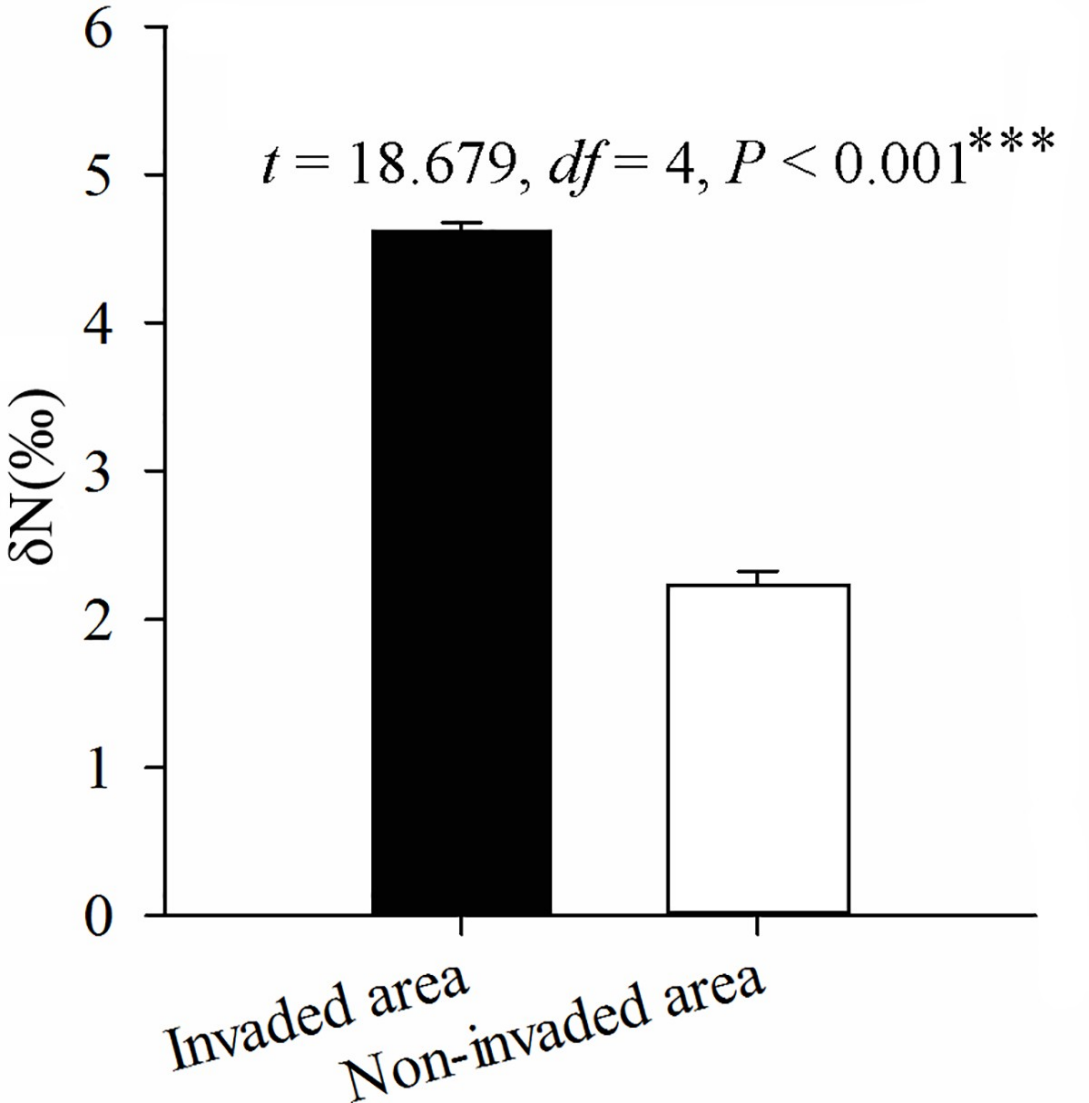
Stable isotope content (δN) (Independent sample t test, mean ± SE, 3 biological replicates were both collected from 3 separate invaded sites and 3 separate non-invaded sites) of *T*. *melanocephalum* workers from *S*. *invicta* invaded and non-invaded areas. Asterisks indicate significant differences (***P < 0.001).

Analysis of stable isotopes of *T*. *melanocephalum* workers from invaded and non-invaded areas showed that workers from invaded areas had significantly higher δN than those from non-invaded areas (Fig 3). Usually, lower δN values are associated with ants primarily feeding on plant-derived diets such as nectar and insect honeydew, while higher δN are found in omnivorous ants [48]. Thus the higher δN value in *T*. *melanocephalum* from invaded areas may suggest that the feeding habits of these ants differ from those in non-invaded areas.

To assess fine-scale geographic variation in stable isotope values across sites, we analyzed three species of annual Asteraceae (including a dominant species, *Bidens pilosa*) occurring as invasive weeds in both invaded and non-invaded areas, and found that these plants did not differ in δN values between these two areas. A fourth species, the invasive annual (or sometimes perennial) herb *Mimosa pudica* (Fabaceae) had significantly lower δN values in invaded sites than in non-invaded sites (S1 Fig). Overall these results, in particular those of the dominant plant, *B*. *pilosa*, suggest that the stable isotope composition of plant communities are similar across the two areas. Thus the different stable isotope signature of *T*. *melanocephalum* in *S*. *invicta* invaded areas seems likely to indicate that colonies have changed their feeding preferences as a consequence of *S*. *invicta* invasion.

### Workers of *T*. *melanocephalum* host significantly different bacterial communities in *S*. *invicta* invaded versus non-invaded areas

The assessment of bacterial titers by qPCR indicated no significant difference in the bacterial content in the gut and gut tissues of *T*. *melanocephalum* workers between colonies from invaded and non-invaded areas. Rarefaction analysis of sequence reads from amplicon sequencing of bacterial 16S rRNA gene showed sufficient depth for analysis (S2 Fig). Of the total 841 bacterial OTUs present in *T*. *melanocephalum* from both areas (only 40 OTUs in invaded areas with abundance above 0.01, versus 32 OTUs in non-invaded areas with abundance above 0.01), 418 OTUs (32 OTUs with abundance above 0.01) including the common insect endosymbiont *Wolbachia* were shared between the bacterial communities of *T*. *melanocephalum* collected from fire ant invaded and non-invaded areas (S2 Fig). This indicates that many OTUs present in *T*. *melanocephalum* from non-invaded areas are also present in *T*. *melanocephalum* from invaded areas. A large number of OTUs in *T*. *melanocephalum* from the invaded areas were unique, while some unique OTUs were also identified in colonies from non-invaded areas (S2 Fig). We compared the alpha diversity (Shannon index) of bacterial communities in each *T*. *melanocephalum* sample, and those from the invaded areas had a lower Shannon index than those from the non-invaded areas (S2 Fig) with no overlap in bacterial communities as shown by distinct clustering patterns in NMDS analysis (stress value = 0.01, S2 Fig). These results indicate that differences in feeding preferences are also correlated with differences in gut microbiota in *T*. *melanocephalum*.

### PICRUSt and LEfSe analyses suggest differences in bacterial symbiont communities

**Fig 4:**
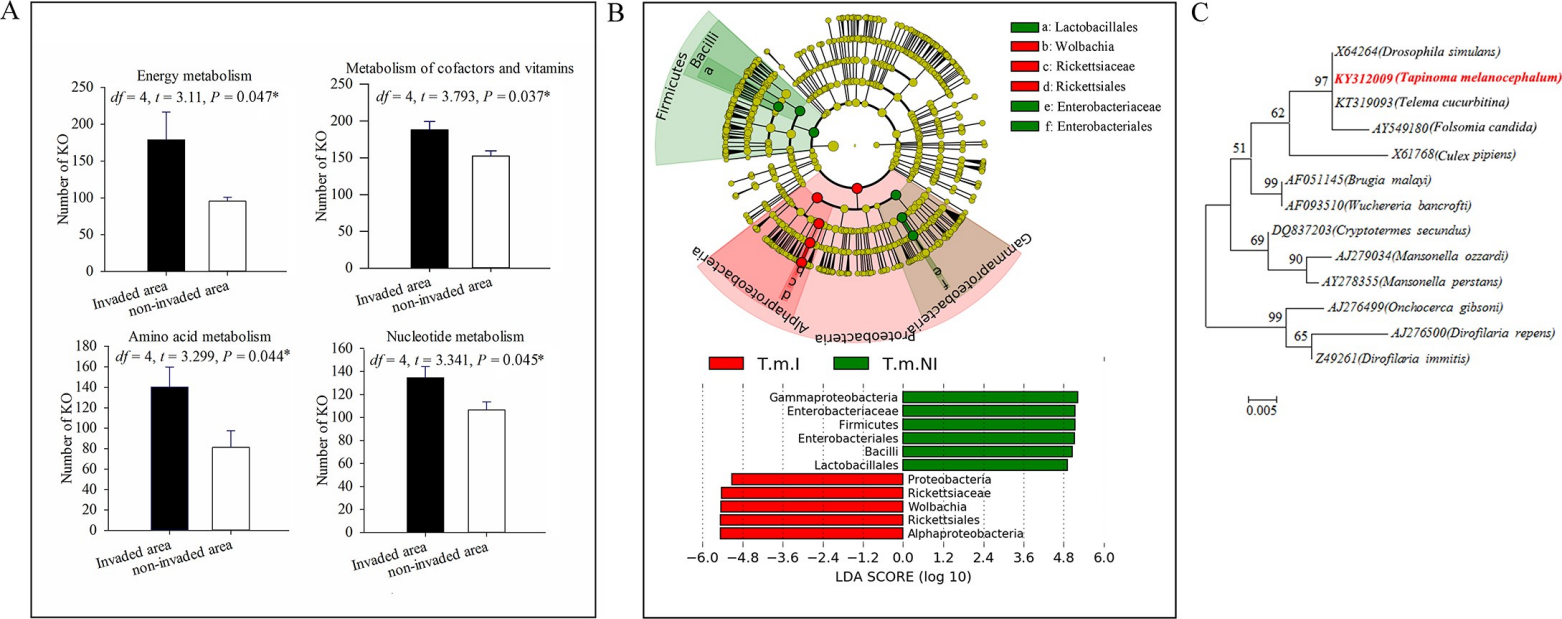
Functional analysis of bacterial symbionts from *T*. *melanocephalum* workers from *S*. *invicta* invaded and non-invaded areas. (A) According to PICRUSt analysis, the most enriched metabolic pathways for symbionts in *T*. *melanocephalum* workers represented as mean number of KO (KEGG orthology) ± SE for energy metabolism, metabolism of cofactors and vitamins, amino acid metabolism and nucleotide metabolism, Independent sample t test, n = 3 biological replicates, P values in the chart were adjusted by a False Discovery Rate (FDR) correction to account for multiple testing [88]). (B) Bacterial groups that differ between *T*. *melanocephalum* workers from *S*. *invicta* invaded and non-invaded areas based on LEfSe analysis. T.m.I: *T*. *melanocephalum* in *S*. *invicta* invaded areas; T.m.NI: *T*. *melanocephalum* in *S*. *invicta* non-invaded areas. (C) V3+V4 areas of the 16S rRNA gene phylogeny of *Wolbachia* from workers of *T*. *melanocephalum* (441 aligned nucleotide sites). Host insect names are in brackets. Bootstrap values (500 replicates) greater than 50% are indicated at the nodes. Sequences of *Wolbachia* collected from *T*. *melanocephalum* workers are in red font. Asterisks indicate significant differences (*P < 0.05).

Functional assignments were predicted from microbial community composition and structure using PICRUSt. Although this analysis, which is based on sequence similarities of the short 16S rRNA gene amplicons needs to be interpreted with caution [49], it revealed potential differences in predicted microbial function across invaded and non-invaded areas. Microbes associated with *T*. *melanocephalum* from invaded areas were different in an array of hypothetical metabolic functions from their counterparts found in non-invaded areas. Pathways for metabolic function, energy metabolism, metabolism of cofactors and vitamins, amino acid metabolism and nucleotide metabolism appeared enriched in *T*. *melanocephalum* from invaded areas (Fig 4A). Amino acid metabolism (that contributes to N cycling) may be the reason for higher δN values detected in *T*. *melanocephalum* from *S*. *invicta* invaded sites.

We also used the LEfSe method to identify bacterial OTUs that were likely to explain most of the differences between the invaded and non-invaded sites. The bacterial orders of OTUs differed between colonies of *T*. *melanocephalum* from the two areas. Alphaproteobacteria were more abundant in *T*. *melanocephalum* from invaded sites, whereas Gammaproteobacteria and Bacilli were more abundant in *T*. *melanocephalum* from non-invaded sites (LDA scores > 4) (Fig 4B). Differences in OTUs mainly spanned two phyla and three classes, with the orders Lactobacillales, Rickettsiales (primarily *Wolbachia*) and Enterobacteriales (primarily Enterobacteriaceae) accounting for the majority of the differences (Fig 4B and S3 Fig). Based on 16S rRNA gene analysis, the *Wolbachia* found in *T*. *melanocephalum* is similar to *Wolbachia* strains found in *Drosophila* fruit flies (Fig 4C).

### Different diets dramatically affect the abundance of *Wolbachia*, Lactobacillales and Enterobacteriaceae in symbiotic microbiota of *T*. *melanocephalum* from invaded and non-invaded areas

**Fig 5:**
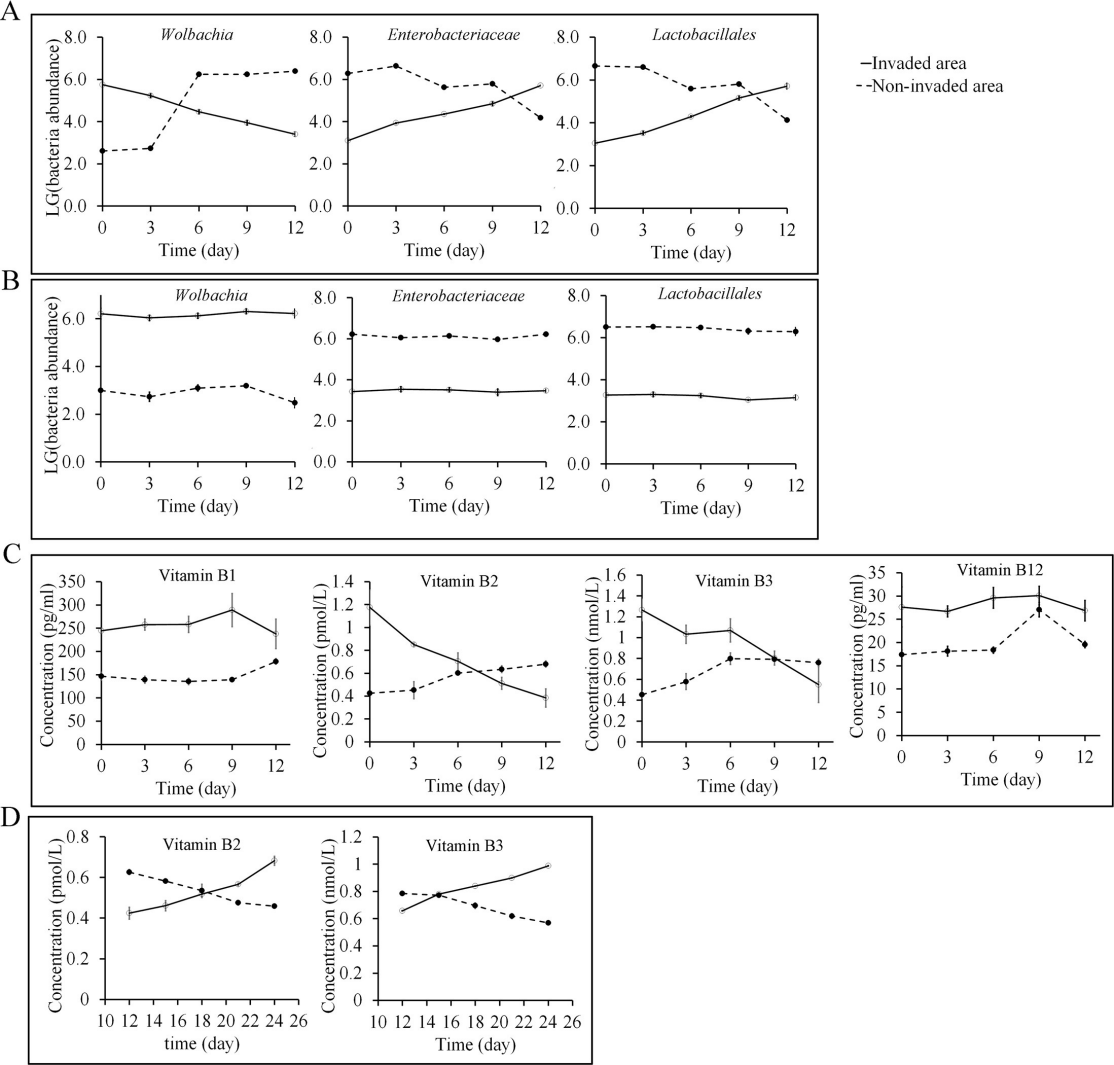
Relative abundances of *Wolbachia*, Lactobacillales and Enterobacteriaceae and vitamin B concentration in workers of *T*. *melanocephalum*. (A) A shift in abundances of *Wolbachia*, Lactobacillales and Enterobacteriaceae in *T*. *melanocephalum* workers from invaded versus non-invaded areas occurs when dietary composition is shifted to sugar (carbohydrate) or locusts (protein) respectively. (B) No shift in abundances of *Wolbachia*, Lactobacillales and Enterobacteriaceae in *T*. *melanocephalum* workers from invaded versus non-invaded areas occurs when diet is not changed in protein or sugar content, respectively. (C) Vitamin B concentration in *T*. *melanocephalum* workers from *S*. *invicta* invaded and non-invaded areas after supplying with sugar and locusts as food, respectively. (D) Vitamin B concentration in *T*. *melanocephalum* workers supplied with complementary food (e.g. peptone or sugar for ants from *S*. *invicta* invaded areas and non-invaded areas, respectively) (as follow-up experiment to (C)). ‘LG’ (on the y-axis) stands for logarithm to the base of 10.

We hypothesized that changes to the food supply of *T*. *melanocephalum* colonies might affect the relative abundances of different bacterial groups. Quantitative PCR assays undertaken with whole ant specimens unveiled a striking reverse effect on the abundance of *Wolbachia*, Lactobacillales and Enterobacteriaceae. *Wolbachia* abundance significantly decreased in workers of *T*. *melanocephalum* from *S*. *invicta* invaded sites (where they feed on a protein-rich diet) with sugar water as a carbohydrate-rich food in the laboratory, while Lactobacillales and Enterobacteriaceae abundances significantly increased (*Wolbachia*: *F*_4,10_ = 125.796, *P* < 0.001; Enterobacteriaceae: *F*_4,10_ = 536.462, *P* < 0.001; Lactobacillales: *F*_4,10_ = 174.642, *P* < 0.001; Fig 5A).

Conversely, *Wolbachia* abundance significantly increased when supplying workers of *T*. *melanocephalum* from *S*. *invicta* non-invaded sites (where they feed on a carbohydrate-rich diet) with only locusts as protein-rich food in the laboratory, while Lactobacillales and Enterobacteriaceae abundances significantly decreased (*Wolbachia*: *F*_4,10_ = 1168.171, *P* < 0.001; Enterobacteriaceae: *F*_4,10_ = 1348.921, *P* < 0.001; Lactobacillales: *F*_4,10_ = 1021.421, *P* < 0.001; Fig 5A).

For the reciprocal controls, colonies of *T*. *melanocephalum* ants from invaded and non-invaded areas were fed locusts and sugar water, respectively, and abundances of *Wolbachia*, Lactobacillales and Enterobacteriaceae were largely unchanged (in non-invaded sites, *Wolbachia*: *F*_4,10_ = 3.47, *P* = 0.0503; Enterobacteriaceae: *F*_4,10_ = 1.019, *P* = 0.443; Lactobacillales: *F*_4,10_ = 0.613, *P* = 0.663; in invaded sites, *Wolbachia*: *F*_4,10_ = 0.543, *P* = 0.708; Enterobacteriaceae: *F*_4,10_ = 0.303, *P* = 0.87; Lactobacillales: *F*_4,10_ = 1.403, *P* = 0.301, Fig 5B). These results indicate that the change in the diet of *T*. *melanocephalum* as a consequence of the presence of *S*. *invicta* affected the relative abundances of *Wolbachia*, Lactobacillales and Enterobacteriaceae in *T*. *melanocephalum*. This change appears to be plastic because it was reversed by supplying ants with different foods.

### Changes in vitamin B content of *T*. *melanocephalum* from *S*. *invicta* invaded and non-invaded sites

*Wolbachia* has been shown to be associated with nutritional roles in other insect species, for example supplementation of B vitamins in bedbugs [50]. We measured the B vitamin contents in workers *T*. *melanocephalum*, and found that the concentrations of vitamin B2 and vitamin B3 were significantly decreased by supplying *T*. *melanocephalum* from *S*. *invicta* invaded areas with sugar (vitamin B2: *F*_4,10_ = 37.942, *P* < 0.001; vitamin B3: *F*_4,10_ = 17.609, *P* < 0.001), while the concentration of vitamin B1 and vitamin B12 were not affected (vitamin B1: *F*_4,10_ = 2.124, *P* = 0.152; vitamin B12: *F*_4,10_ = 1.118, *P* = 0.401; Fig 5C).

The concentrations of vitamin B2 and vitamin B3 were significantly increased by supplying workers of *T*. *melanocephalum* from *S*. *invicta* non-invaded areas with locusts (vitamin B2: *F*_4,10_ = 27.355, *P* < 0.001; vitamin B3: *F*_4,10_ = 32.297, *P* < 0.001, Fig 5C). Concentrations of vitamin B2 and vitamin B3 could be recovered after the ants were fed with complementary food (sugar for the ants from *S*. *invicta* invaded areas and peptone for the ants from *S*. *invicta* non-invaded areas) (ants from *S*. *invicta* invaded area: vitamin B2: *F*_4,10_ = 22.492, *P* < 0.001; vitamin B3: *F*_4,10_ = 14.523, *P* < 0.001; ants from *S*. *invicta* non-invaded area: vitamin B2: *F*_4,10_ = 17.993, *P* < 0.001; vitamin B3: *F*_4,10_ = 43.626, *P* < 0.001; Fig 5D). The concentrations of vitamin B2 and vitamin B3 were not significantly affected by supplying the workers of *T*. *melanocephalum* from invaded and non-invaded areas with locusts and sugar, respectively (*P* > 0.05 for all, S4 Fig).

### The abundance of *Wolbachia* in the ant microbiome is positively correlated with concentrations of vitamin B2 and B3 in workers of *T*. *melanocephalum* in *S*. *invicta* non-invaded areas

The concentrations of vitamin B2 and vitamin B3 in workers increased or decreased depending upon the abundance of *Wolbachia*. *Wolbachia* abundance was positively correlated with vitamin concentrations identified in *T*. *melanocephalum* in *S*. *invicta* non-invaded areas: *Wolbachia* abundance versus vitamin B2: R = 0.99, *P* = 0.001; *Wolbachia* abundance versus vitamin B3: R = 0.898, *P* = 0.039; in *S*. *invicta* invaded areas: *Wolbachia* abundance versus vitamin B2: R = 0.924, *P* = 0.025) expect vitamin B3 in *T*. *melanocephalum* from *S*. *invicta* invaded areas (R = 0.756, *P* = 0.14).

### Data from additional sites further confirmed differences in metabolism and bacterial symbiont communities of *T*. *melanocephalum* between in *S*. *invicta* invaded and non-invaded areas

**Fig 6:**
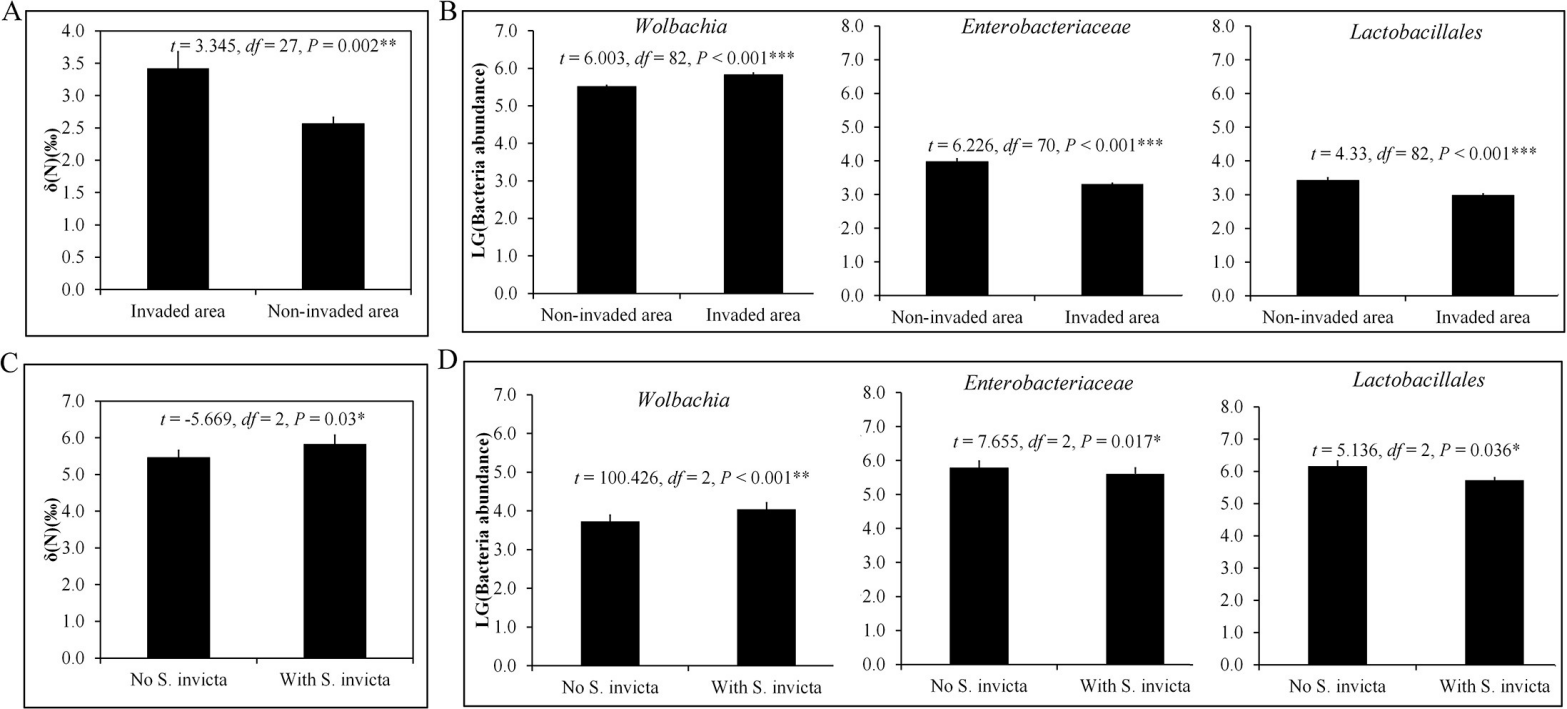
Stable isotope content δN and abundances of *Wolbachia*, Lactobacillales and Enterobacteriaceae in more wild populations of *T*. *melanocephalum*. (A) Comparison for the δN values (Independent sample t test, mean ± SE) and (B) abundances of *Wolbachia*, Lactobacillales and Enterobacteriaceae (Independent sample t test, mean ± SE) in *T*. *melanocephalum* from another three non-invaded and four invaded sites. (C) Comparison for the δN values (Paired sample t test, mean ± SE, n = 3 biological replicates) and (D) abundances of *Wolbachia*, Lactobacillales and Enterobacteriaceae (Paired sample t test, mean ± SE, n = 3 biological replicates) in *T*. *melanocephalum* after rearing with or without *S*. *invicta* for one month. ‘LG’ (on the y-axis) stands for logarithm to the base of 10. Asterisks indicate significant differences (*P < 0.05, **P < 0.01, ***P < 0.001).

The functional relationships of *T*. *melanocephalum* in the presence or absence of fire ants was assessed for seven further field sites that are more distant from each other than the core study sites. These additional field data showed that δN values of *T*. *melanocephalum* from four additional invaded sites (13 colonies) were significantly higher than those from three additional non-invaded sites (16 colonies) (Fig 6A). qPCR assays also revealed that the abundance of *Wolbachia* was significantly higher in workers from invaded areas than non-invaded areas (Fig 6B) and the abundance of Lactobacillales and Enterobacteriaceae was significantly higher in workers from non-invaded areas than invaded areas (Fig 6B). To directly test the effects of *S*. *invicta* on *T*. *melanocephalum's* diet and microbiota, we collected three *T*. *melanocephalum* colonies from the wild and reared them in the lab either with or without *S*. *invicta* for one month. We then measured δN as well as abundances of *Wolbachia*, Lactobacillales, and Enterobacteriaceae. The δN values in *T*. *melanocephalum* were significantly increased after being reared in competition with *S*. *invicta* (Fig 6C) and the abundances of *Wolbachia*, Lactobacillales and Enterobacteriaceae were significantly affected (Fig 6D).

## Discussion

Our study provides evidence that invasion by *S*. *invicta* may change the composition of the resident ant community. We tested this indirectly by comparing invaded and non-invaded sites since we were not allowed to infest uninvaded areas with *S*. *invicta* to elucidate this problem directly, and, therefore, other factors may also contribute to the findings. Some ant species do not exist in *S*. *invicta* invaded areas, whereas others, particularly *T*. *melanocephalum* have significantly higher numbers. This effect may result in part from reduced competition between *T*. *melanocephalum* and other ant species following *S*. *invicta* invasion [51]. However, *T*. *melanocephalum* workers inhabiting fire ant invaded areas also displayed submissive and avoidance behaviors when attacked by *S*. *invicta* workers. Similar behavioral or morphological changes can be found in other ants [52], frogs [53], plants [54], marine animals [55] and other terrestrial animals [56].

We also found that the stable isotope composition of *T*. *melanocephalum* workers differed between colonies located in the invaded and non-invaded areas. For δC, no differences were observed, while significant differences were detected for δN. Thus the higher δN value in *T*. *melanocephalum* from invaded areas suggests that the feeding habits of these ants may have changed as a consequence of the *S*. *invicta* invasion. To persist, *T*. *melanocephalum* may settle for different diets to avoid interspecific competition with *S*. *invicta* for nectar and insect honeydew. More variable food resources are expected to result in greater variation of δC values [57], and no significant difference was detected in δC for *T*. *melanocephalum* from the two different areas.

In addition to diet differences, we found systematic differences in the composition and structure of bacterial symbiont communities in *T*. *melanocephalum* workers collected from *S*. *invicta* invaded and non-invaded areas. Although *Wolbachia* was present in both invaded and non-invaded sites, we found a larger number of *Wolbachia* sequence reads in *T*. *melanocephalum* workers from *S*. *invicta* invaded sites. *Wolbachia* are intracellular bacteria that exist mainly in the reproductive tissues (testis and ovary) of arthropod hosts so that they can manipulate the reproduction of their hosts. In various insects, *Wolbachia* has also been detected in other tissues, including in gut epithelial tissues and in accessory digestive glands such as salivary glands. *Wolbachia* has also been found to be important to fitness in some host species, and *Wolbachia* titres can increase in some stressed insects [58]. *Wolbachia* has been shown to be associated with nutritional roles in other insect species, e.g. supplementation of B vitamins in bedbugs [50]. In *Drosophila*, *Wolbachia* was shown to improve host fitness via metabolic provisioning during periods of nutritional stress [59]. Based on 16S rRNA gene analysis, the *Wolbachia* found in *T*. *melanocephalum* is similar to *Wolbachia* strains found in *Drosophila* fruit flies. Difference in *Wolbachia* titers may be linked to changes in B vitamin metabolism in *T*. *melanocephalum* from *S*. *invicta* invaded areas. Usually ants are attracted to feed on nectar and insect honeydew, which have high sugar content, and studies have indicated that Lactobacillales and Enterobacteriales have strong abilities to decompose sugar [60–62]. The observation of a higher abundance of Lactobacillales and Enterobacteriaceae in *T*. *melanocephalum* from *S*. *invicta* non-invaded areas is consistent with easier access to nectar and insect honeydew.

Bacteria such as *Wolbachia*, Lactobacillales and Enterobacteriaceae have been identified by 16S rRNA gene amplicon sequencing studies in a variety of ants [63–65]. Different hypotheses have been investigated regarding their possible functions in the host, particularly those related to nitrogen metabolism [66]. Our experiments further indicate that the difference in relative abundances of *Wolbachia*, Lactobacillales and Enterobacteriaceae between *T*. *melanocephalum* from *S*. *invicta* invaded areas and non-invaded areas can be affected by the type of food available to colonies of *T*. *melanocephalum*. This is consistent with results from other studies showing that *Wolbachia* titers can be affected by diet [67–69], and that microorganisms are important for B vitamin metabolism in insects [70]. For example, high larval mortality occurred in species of lice in the genus *Pediculus* when symbiotic bacteria were removed, but this effect was reduced when the diet was supplemented with vitamin B3 (nicotinic acid) [71]. In aphids, symbionts have been shown to possess the biosynthetic pathway for the synthesis of vitamin B2 (riboflavin) [72]. The role in riboflavin synthesis has been supported by dietary experiments and genomic data are consistent with the expectation that microorganisms play a role in B vitamin provisioning to aphids [73].

The ecological effects of biological invasions have been well studied [74]. However, few investigations have examined the likely ecological and behavioral change to invasions by native and other co-existing resident species. In our study, we demonstrated that host–symbiont interactions of resident species may change in response to biological invasions, and that symbiotic bacteria may play a role in the adaptation of resident host taxa to invasive species. We found that bacterial communities associated with workers of *T*. *melanocephalum* in *S*. *invicta* invaded areas were distinct from those of *T*. *melanocephalum* in *S*. *invicta* non-invaded areas. However, additional genetic and/or functional studies of associated microbiota, such as experimental manipulation of ant communities in the field (to overcome spatial issues), are required to fully understand the microbial-host interactions that occur in *T*. *melanocephalum* following *S*. *invicta* invasion. We also found that the titer of *Wolbachia* is dependent on the nutritional status of its host. This has previously been observed for *Drosophila* [68] and is an important finding in the context of pest management strategies that rely on *Wolbachia*.

## Methods

### Quantifying ant abundance across *S*. *invicta* invaded and non-invaded areas

Our field experiments and sample collections were carried out in three *S*. *invicta* invaded and three non-invaded sites in Guangzhou, China (S5 Fig). At each site, one plot (approximate 1,000 m^2^) was randomly selected for further investigation and sampling. This region has a humid, subtropical, monsoon climate, with 1,696 mm of annual rainfall, a minimum monthly average temperature of 21.9°C in January, and a maximum of 28.4°C in July [75]. The invaded and non-invaded sites are more than 3 km apart, and our continuous observations over more than five years confirmed that they are in zones that have either been invaded by *S*. *invicta*, or not been invaded by *S*. *invicta*. Significant genotypic differentiation of workers between sites demonstrate that the invaded and non-invaded sites contain different *T*. *melanocephalum* colonies [76]. The study areas have not been used for farming for more than five years [77] but carry many weeds, and are dominated by the weed *Bidens pilosa* L.

To evaluate the potential impact of *S*. *invicta* invasion on the diversity of resident ant communities, ants were sampled from each plot between September and October 2015. Pitfall trapping and baiting were used to sample the ants in three invaded and three non-invaded sites. For trapping, a 100 mL centrifuge tube containing 40 ml of 45% alcohol was buried so that the opening of the tube was flush with the ground surface. In each plot, three traps were randomly set and left for 24 h. For baiting, ham sausage and honey were placed in a 30 ml transparent plastic bottle that was placed horizontally on the ground for 30–60 min. In each plot, three baits were randomly set between 8:00–18:00 h. The Simpson index (*C*), Shannon-Wiener index (*H’*) and Pielou evenness index (*E*) were calculated and compared between fire ant invaded and non-invaded areas.

### Aggressive behavior between *T*. *melanocephalum* and *S*. *invicta*

To determine whether coexisting colonies of *T*. *melanocephalum* change their behavior in response to fire ant invasion, individuals were collected from *T*. *melanocephalum* colonies in the *S*. *invicta* non-invaded sites (2 colonies were collected from GZ1, 3 colonies from GZ2, S5 Fig), and individuals of *S*. *invicta* and *T*. *melanocephalum* colonies from the *S*. *invicta* invaded sites (all colonies were collected from GZ4, S5 Fig). The collected colonies were maintained in plastic nest boxes whose walls had been painted with Fluon in a temperature controlled room at 26°C and 80% humidity, and provided with sugar water (20% w/v) and locusts, *Locusta migratoria manilensis* (Meyen) as food every day. After rearing for one month, the ants were used for experiments.

We quantified interspecific aggression between the two ant species using the following behavioral assay adapted from a previous report [78]. To test for interspecific individual aggression, one medium-sized (length, 4–5 mm) *S*. *invicta* worker and one *T*. *melanocephalum* worker were placed in a Petri dish (diameter = 4.0 cm, height = 1.5 cm) using a brush. Interactions were scored on a scale from 1 to 4, following protocols from an earlier study [52] and adapted for fire ants in this study: ants exhibited no change in direction or posture upon encounter or turned and moved away (Level I), ants made antennal contact that lasted for more than one second (Level II), ants opened their mandibles or turned their gasters upwards or towards their heads (Level III), both ants attacked each other and were twisted together, or one ant fiercely attacked the other with upper jaws grappling or stinging (Level IV). After five minutes, the ants’ attack scores and times were recorded, and an aggressiveness index calculated using the following formula $\frac{\sum_{i=1}^{n}\delta_{i}f_{i}}{T}$ for each trial [52]. In this formula, *δ*_i_ and *f*_i_ are the interaction score and frequency of each act, respectively, and *T* is the total interaction frequency, which is defined as the sum of all contacts between ants. Five pairs of colonies were tested. Ten trials, each involving different workers, were conducted for each pair of colonies.

For group aggression experiments, ten medium-sized (length, 4–5 mm) workers of *S*. *invicta* and ten *T*. *melanocephalum* workers from colonies of fire ant invaded and non-invaded sites were randomly selected and placed in a Petri dish (diameter = 9 cm, height = 1.5 cm, sides coated with Fluon) using a brush. Mortality was recorded after 0.5 h, 1 h, 2 h and 4 h. Five pairs of colonies were tested, and three trials, each involving different worker samples, were conducted for each pair of colonies. Ants whose bodies were so damaged that they could not stand after the encounter were considered dead.

### Behavioral adaptation of *T*. *melanocephalum* to the attack of *S*. *invicta*

To determine whether contact experience between *S*. *invicta* and *T*. *melanocephalum* influenced levels of aggression between the two species, a single worker of *T*. *melanocephalum* collected from the non-invaded area (workers from 2 colonies were collected at GZ1, and from 3 colonies at GZ2, S5 Fig) was placed in the Petri dish, and 1 min later, a worker of *S*. *invicta* (collected from GZ4, S5 Fig) was introduced to the same Petri dish. We removed the *S*. *invicta* worker when it became apparent that the two workers were going to fight. At intervals of 0.5 h, 1 h, 2 h or 4 h later, we introduced another *S*. *invicta* worker and again tested the level of interspecific individual aggression for 5 min. Fifteen pairs of colonies were tested. Three trials, each involving different workers, were conducted for each pair of colonies.

### Stable isotope analysis

In order to investigate whether *S*. *invicta* invasion could affect the feeding habits of *T*. *melanocephalum*, the stable isotope composition of *T*. *melanocephalum* workers from both invaded and non-invaded sites was assessed. Workers of *T*. *melanocephalum* were collected from three sites within the area invaded by *S*. *invicta* (sites GZ4, 5, 6, S5 Fig), and three sites within the area not invaded by *S*. *invicta* (sites GZ1, 2, 3, S5 Fig) between September and October 2015. For each site, 200 ant workers were collected, pooled together and stored at -80°C. We removed the gasters from each worker of *T*. *melanocephalum* to prevent recent stomach contents from influencing δ^15^ N values [79]. The same species of plants (i.e. *Bidens pilosa*, *Mimosa pudica*, *Ageratum conyzoides* and *Erigeron canadensis*) from invaded and non-invaded areas were collected between September and October 2015. Three young leaves for each plant and eight plants from each site were randomly collected. We dried all samples at 60°C for 24–48 h. To prepare samples for isotopic analysis, samples were ground with a mortar and pestle, and 1 mg of each sample was packed into a tin capsule.

An Isotope Ratio Mass Spectrometer (Thermo Fisher Scientific, Inc., USA) was used to measure stable isotopes according the manufacturer’s instructions. Stable isotope abundance (δ) was calculated as follows [79]:

$$\delta (‰)=\left(\frac{R_{Sa}}{R_{St}}-1\right)*1000;$$

R_Sa_ is the detected value of the collected samples; R_St_ is the detected value of the standard sample.

To determine whether the fine-scale geographic distribution of stable isotopes was the same between the invaded and non-invaded sites, the stable isotope composition of plants was compared.

### Response of *T*. *melanocephalum* bacterial symbionts to *S*. *invicta* invasion

Bacterial symbiont communities of *T*. *melanocephalum* in the *S*. *invicta* invaded sites (sites GZ4, 5, 6, S5 Fig) were compared with those in the non-invaded sites (sites GZ1, 2, 3, S5 Fig) and the potential function of the symbionts was investigated.

For each site, 100 ant workers were randomly collected from at least 10 colonies. Colony boundary aggression tests suggested that all 10 colonies were separate colonies [76]. Guts of workers from each site were pooled together in pure alcohol and stored at -80C. For each site, 100 worker guts were transferred into centrifuge tubes containing DNA extraction buffer. DNA was extracted using a DNA extraction kit (Tiangen biotech CO., LTD, Beijing, China) following the manufacturer’s instructions. The bacterial 16S rRNA gene was amplified from the extracted DNA by PCR using two primers targeting the V3+V4 variable region of the 16S rRNA gene (16S-F: 5’-CCTACGGGNGGCWGCAG-3’, 16S-R: 5’-GGACTACHVGGGTATCTAAT-3’) [80]. qPCR was used to estimate the absolute content of bacterial DNA in the next generation sequencing samples by using universal bacterial 16S rRNA gene primers (see below). A standard curve for qPCR was generated by amplifying a 16S rRNA gene fragment of *E*.*coli*. Each sample was analyzed in a total reaction volume of 25 μL containing 2.5 μL of Takara 10× Ex Taq buffer, 1.5 μL of Mg^2+^ (25 mM), 2 μL of dNTPs (2.5 mM), 0.25 μL of Takara Ex Taq (2.5 U/μL), 0.5 μL of each primer (10 μM), 16.75 μL of ddH_2_O and 25ng of template. Three PCR amplifications for each sample were performed with a 2 min incubation at 95C followed by 30 cycles at 94C for 30 s, 57C for 30 s, and 72C for 30 s, with a final 5 min extension at 72C. Each set of experiments included negative controls with sterile distilled water instead of template DNA. No amplified products were found in the negative controls. The PCR products were purified using a QIAGEN MinElute PCR Purification Kit to remove unincorporated primers and nucleotides. A micro-spectrophotometer ND-1000 (NanoDrop Technologies, Wilmington, DE, USA) was used to measure the concentration of the purified DNA. Adapters were added to the purified DNA to build a library for sequencing using the Illumina sequencing kit (www.illumina.com/company/legal.html) and an Illumina MiSeq sequencer (Illumina, San Diego, CA, USA). Amplicons were then pooled in equimolar fashion and paired-end sequenced (2 × 250) on an Illumina platform according to the standard protocols. For each sample, more than 50,000 reads were obtained. After sequencing, the data were filtered to remove reads containing more than 10% of unresolved nucleotides (N) and reads containing less than 80% of bases with a Q-value > 20. Paired end clean reads were merged as raw tags using FLSAH (v 1.2.11) with a minimum overlap of 10bp and mismatch error rates of 2%. Noisy sequences of raw tags were filtered by QIIME (V1.9.1) pipeline under specific filtering conditions to obtain the high-quality clean tags. Clean tags were searched against the reference database (http://drive5.com/uchime/uchime_download.html) to perform reference-based chimera checking using UCHIME algorithm (http://www.drive5.com/usearch/manual/uchime_algo.html). All chimeric tags were removed and finally obtained effective tags for further analysis. To obtain unique tags and to determine the number of tags in the dataset, the dataset was subjected to redundancy treatment using Mothur software (v. 1.27.0) [81]. Moreover, rarefaction curves were calculated by Mothur for all samples to evaluate the sequencing saturation. The representative sequences were classified into organisms by naïve Bayesian model using RDP classifier [82] based on GreenGene Database (http://greengenes.lbl.gov/cgi-bin/nph-index.cgi). The tags were clustered into operational taxonomic units (OTUs) at ≥ 97% similarity using the UPARSE [83] pipeline. The tag sequence with highest abundance was selected as representative sequence within each cluster. The species annotations and abundance information of the OTUs were used to generate OTU abundance profiles for all samples. To determine the bacterial taxa that most likely explained differences between sites, we used the linear discriminant analysis (LDA) effect size (LEfSe) method (http://huttenhower.sph.harvard.edu/galaxy/) [84]. Metagenomic data would be the best option for functional evaluation [85], but unfortunately such data sets are limited. Albeit there is a limited predictive power of 16S rRNA gene diversity for function of insect associated symbionts, we have used PICRUSt [49,86] to explore putative functions and pathways of OTUs against the KEGG database. Briefly, OTUs were normalized by copy number, and the gene categories were predicted at level 2 and level 3 KEGG orthology groups (KOs). The metagenomic prediction can produce the KEGG IDs and Enzyme Commission IDs. Unweighted unifrac distance matrix was generated by QIIME. Non-metric multidimensional scaling (NMDS) of unweighted Unifrac distances was calculated for the OTUs at phylum level and plotted in R with Welch's t-test. Ordination was done in two dimensions. Ten iterations were performed, and the iteration resulting in the lowest stress was plotted. The 16S rRNA gene sequencing data was deposited in TSA database of NCBI (Accession number: PRJNA496064)

### Phylogenetic analysis of *Wolbachia*

The 16s rRNA gene sequence of the most abundant *Wolbachia* OTU was submitted to BLAST in NCBI (https://blast.ncbi.nlm.nih.gov/Blast.cgi). With the top hits in BLAST, a neighbor-joining phylogenetic analysis was performed with MEGA 5.0 [87]. The neighbor-joining (NJ) method was used to construct a phylogenetic tree based on the sequence of 16S rRNA gene and the phylogenetic tree was evaluated by Bootstrap analysis.

### Effect of diet difference on bacterial abundance in *T*. *melanocephalum* from *S*. *invicta* invaded and non-invaded sites

To investigate whether symbiotic bacteria of *T*. *melanocephalum* workers were different between *S*. *invicta* invaded and non-invaded areas, 12 individual colonies of *T*. *melanocephalum* from *S*. *invicta* invaded and non-invaded sites were collected (2 colonies were collected from each site of GZ1-6 and were kept in boxes in the laboratory, S5 Fig). Colony boundary aggression tests suggested that all 12 colonies were separate colonies [76]. The experimental design was as follows: for *S*. *invicta* non-invaded sites three colonies were fed with sugar (a carbohydrate-rich diet), and three were fed with locusts (a protein-rich diet); for *S*. *invicta* invaded sites three colonies were fed with sugar, and three colonies were fed with locusts. Workers were randomly sampled from these colonies every 3 days for 12 days. The DNA of 15 workers per colony was extracted as one pooled sample using a DNA extraction kit (Tiangen, Beijing, China) following the manufacturer’s instructions. The absolute abundances of *Wolbachia*, Enterobacteriaceae and Lactobacillales bacteria were measured by real-time fluorescent quantitative PCR with designed specific 16S rRNA gene primers for *Wolbachia* (F: GCTGCAGTGGGGAATATTGG; R: TAACGCTAGCCCTCTCCGTA), Enterobacteriaceae (F: TATTGCACAATGGGCGCAAG; R: GGAGTTAGCCGGTGCTTCTT) and Lactobacillales (F: TATTGCACAATGGGCGCAAG; R: GGAGTTAGCCGGTGCTTCTT). Quantitative PCR analyses were performed for individuals that were fed different diets. PCR analyses were conducted on an Agilent Technologies Stratagene M×3005P by real-time quantitative PCR. Each treatment was measured in three separate technical replicates with a total reaction volume of 25 μL containing 0.5 μL of each primer (diluted to 10 mM), 12.5 μL SYBR Premix Ex Taq^TM^, 9.5 μL ddH2O and 2 μL template. Cycling conditions were as follows: 95°C for 10 min and 40 cycles of 95°C for 30 s, 60°C for 45 s and 72°C for 1 min. A standard curve for qPCR was generated by amplifying the 16S rRNA gene of *Escherichia coli* as a representative bacterium of the family Enterobacteriaceae. For this purpose, *E*. *coli* was inoculated in LB liquid medium and cultivated for 2 days. Then, Colony-Forming Units (CFUs) of *E*.*coli* were calculated with a blood counting chamber. The DNA of *E*. *coli* was extracted from 2 ml culture. The extracted DNA was diluted (1×, 0.1×, 0.01×, 0.001×) and the DNA dilution series was submitted to 16S rRNA gene qPCR. For each concentration, 3 replicates were done.

### B vitamin content of workers from colonies of *T*. *melanocephalum* living in *S*. *invicta* invaded and non-invaded areas following food supplementation

To test whether availability and type of food source might affect colonies of *T*. *melanocephalum* inhabiting *S*. *invicta* invaded and non-invaded areas, workers collected in the previous step (workers from 2 colonies were collected from each site of GZ1-6, S5 Fig) were also sent to test the contents of vitamin B1, vitamin B2, vitamin B3 and vitamin B12 using an enzyme-linked immunosorbent assay (ELISA) kit (Shanghai, China, http://www.mlbio.cn/?bdmlbio-12) according to the manufacturer’s instructions. For each of these colonies, 15 workers were randomly collected to measure their content of B vitamins. Moreover, the colonies fed with sugar for 12 days were provided with peptone as an alternative protein food source (to demonstrate that changes in abundance of *Wolbachia* and other bacterial DNA is not due to locust-associated bacteria when *T*. *melanocephalum* is feeding on locust), and the colonies fed with locusts for 12 days were provided with sugar, for another 12 days. Then the ants were sampled every 3 days. After the samples were collected, the content of vitamin B1 and vitamin B2 was measured. The content of vitamin B1 and vitamin B2 was also measured after the ants collected from the invaded sites and non-invaded site were fed with locusts and sugar, respectively.

### Further field and laboratory experiments to test the response of bacterial symbionts of *T*. *melanocephalum* to *S*. *invicta* invasion

To further confirm the responses of bacterial symbionts of *T*. *melanocephalum* to *S*. *invicta* invasion, *T*. *melanocephalum* from another three non-invaded sites (16 colonies) and four invaded sites (13 colonies) were collected (colony boundary aggression tests suggested that all colonies were separate colonies, S6 Fig). Then, the δN values and the abundance of *Wolbachia*, Enterobacteriaceae and Lactobacillales were compared. To further confirm the different trophic patterns of *T*. *melanocephalum* in invaded and non-invaded areas are caused by the competition of *S*. *invicta*, we performed the competition tests in laboratory in order to control for spatial factors. Three colonies of *T*. *melanocephalum* (300 workers for each colony) from non-invaded sites were collected. The experiment was run in boxes divided into three rooms, two smaller rooms and one larger room (S7 Fig). One colony of *T*. *melanocephalum* and one colony of *S*. *invicta* (100 workers) were placed into the two smaller rooms. Meanwhile, sugar (a carbohydrate-rich diet) and locusts (a protein-rich diet) were randomly placed into the larger room. Ants were given access to either rooms via holes in the room walls. In this way, we were able to evaluate the impact of competitive pressure from *S*. *invicta* on the trophic patterns of *T*. *melanocephalum*. As control, *T*. *melanocephalum* was reared in a two room box (*T*. *melanocephalum* was placed in one room, sugar and locusts were randomly placed into the other room). In this case, we were able to simulate the situation of *T*. *melanocephalum* without the competition from *S*. *invicta*. After the colonies were reared for one month, the δN values (15 workers per sample) and the abundance of *Wolbachia*, Enterobacteriaceae and Lactobacillales were compared between treatment and control.

### Statistical analysis

Ant species diversity indices (*C*, *H’*, *E*) were calculated for all samples. Independent sample t tests were used to compare alpha diversity values and δN values between *T*. *melanocephalum* in invaded areas and non-invaded areas. One-way analysis of variance (ANOVA) followed by Tukey’s test for multiple comparisons was used to compare the bacterial abundance and vitamin B contents in *T*. *melanocephalum* after reared with different diets. δ^15^N values and the abundance of *Wolbachia*, Enterobacteriaceae and Lactobacillales in *T*. *melanocephalum* after rearing with or without *S*. *invicta* for one month were compared with paired sample t tests. For 16S rRNA gene sequences, we used the linear discriminant analysis (LDA) effect size (LEfSe) method.

## Supporting information

S1 Fig Stable isotope content (mean ± SE) (δN) of plants in *S*. *invicta* invaded and non-invaded areas. (TIF)

S2 Fig **Community structure of bacterial symbionts from workers of *T*. *melanocephalum* in *S*. *invicta* invaded and non-invaded areas. Shared and unique OTUs in bacterial symbionts from workers of *T*. *melanocephalum* from the two areas** (A); Rarefaction analysis based on the Shannon index for bacterial symbionts from workers. The index values are shown on the y-axis and the number or reads sampled are shown on the x-axis (B). Alpha diversity (Shannon index) (mean ± SE) of bacterial symbionts from workers of *T*. *melanocephalum* from the two areas (C); NMDS plot analyses based on Bray Curtis distances. T.m.I: *T*. *melanocephalum* in *S*. *invicta* invaded areas (D); T.m.NI: *T*. *melanocephalum* in *S*. *invicta* non-invaded areas.
(TIF)

S3 Fig Relative abundance (mean ± SE) of Bacillales, Lactobacillales, *Wolbachia* and Enterobacteriaceae of *T*. *melanocephalum* in *S*. *invicta* invaded and non-invaded areas as collected in the field (without experimental feeding in the laboratory). (TIF)

S4 Fig Vitamin B2 and B3 concentration in workers of *T*. *melanocephalum* from *S*. *invicta* invaded and non-invaded areas after supplying with locusts and sugar as food, respectively (in invaded area, vitamin B2: *F*_4,10_ = 0.595, *P* = 0.674; vitamin B3: *F*_4,10_ = 1.697, *P* = 0.227; in non-invaded area, vitamin B2: *F*_4,10_ = 0.244, *P* = 0.907; vitamin B3: *F*_4,10_ = 0.888, *P* = 0.505). (TIF)

S5 Fig Locations of the three *S*. *invicta* invaded (red dots) and three non-invaded (black dots) sites in this study. The map for study sites was adapted from the Wikipedia website (https://en.wikipedia.org/wiki/File:Guangzhou_city_map_plan_China_Level_12_English.svg, https://countrydigest.org/guangzhou-population/, https://upload.wikimedia.org/wikipedia/commons/3/31/Guangdong_administrative_divisions_2009_1level-fr.svg).
(TIF)

S6 Fig Locations of the four *S*. *invicta* invaded (red dots) and three non-invaded (black dots) sites in this study. The map for study sites was adapted from the website (https://en.wikipedia.org/wiki/Guangdong, http://d-maps.com/m/asia/china/guangdong/guangdong19.gif).
(TIF)

S7 Fig Boxes used for examining the effect of *S*. *invicta* invasion on *T*. *melanocephalum*. Left: treatment; Right: control.
(TIF)

S1 Table Number of ants per species captured in *S*. *invicta* invaded and non-invaded area. (DOCX)

## References

[1] Early R, Bradley BA, Dukes JS, Lawler JJ, Olden JD, et al (2016) Global threats from invasive alien species in the twenty-first century and national response capacities. Nature Communications 7: 12485 10.1038/ncomms12485 27549569 PMC4996970

[2] Hierro JL, Maron JL, Callaway RM (2005) A biogeographical approach to plant invasions: the importance of studying exotics in their introduced and native range. Journal of Ecology 93: 5–15.

[3] Richardson DM, Pysek P (2012) Naturalization of introduced plants: ecological drivers of biogeographical patterns. New Phytologist 196: 383–396. 10.1111/j.1469-8137.2012.04292.x 22943470

[4] Traveset A, Richardson DM (2014) Mutualistic Interactions and Biological Invasions. Annual Review of Ecology, Evolution, and Systematics 45: 89–113.

[5] Yu FK, Huang XH, He Y, Fu DG, Liu CE, et al (2014) Growth disturbance of extracts from several crops straw (residue) on Ageratina adenophora and biological-control implications in hazardous weed invasion for eco-restoration. Ecological Engineering 63: 127–133.

[6] Yu FK, Huang XH, Duan CQ, He SZ, Zhang GS, et al (2014) Impacts of Ageratina adenophora invasion on soil physical-chemical properties of Eucalyptus plantation and implications for constructing agro-forest ecosystem. Ecological Engineering 64: 130–135.

[7] Suarez AV, McGlynn TP, Tsutsui ND (2009) Biogeographic and Taxonomic Patterns of Introduced Ants. Campmadron Org: 233–244.

[8] Ross KG (1994) Exotic ants—biology, impact, and control of introduced species. Science 265: 1744–1745. 10.1126/science.265.5179.1744 17770900

[9] Morrison LW (2000) Mechanisms of interspecific competition among an invasive and two native fire ants. Oikos 90: 238–252.

[10] Hook AW, Porter SD (1990) Destruction of harvester ant colonies by invading fire ants in south-central TEXAS (Hymenoptera, Formicidae). Southwestern Naturalist 35: 477–478.

[11] Strauss SY, Lau JA, Carroll SP (2006) Evolutionary responses of natives to introduced species: what do introductions tell us about natural communities? Ecology Letters 9: 354–371. 10.1111/j.1461-0248.2005.00874.x 16958902

[12] Chivers DP, Wildy EL, Kiesecker JM, Blaustein AR (2001) Avoidance response of juvenile pacific treefrogs to chemical cues of introduced predatory bullfrogs. Journal of Chemical Ecology 27: 1667–1676. 11521404 10.1023/a:1010418526991

[13] Cousyn C, De Meester L, Colbourne JK, Brendonck L, Verschuren D, et al (2001) Rapid, local adaptation of zooplankton behavior to changes in predation pressure in the absence of neutral genetic changes. Proceedings of the National Academy of Sciences of the United States of America 98: 6256–6260. 10.1073/pnas.111606798 11353872 PMC33455

[14] Bourke P, Magnan P, Rodriguez MA (1999) Phenotypic responses of lacustrine brook charr in relation to the intensity of interspecific competition. Evolutionary Ecology 13: 19–31.

[15] Filchak KE, Roethele JB, Feder JL (2000) Natural selection and sympatric divergence in the apple maggot *Rhagoletis pomonella*. Nature 407: 739–742. 10.1038/35037578 11048719

[16] Huttenhower C, Gevers D, Knight R, Abubucker S, Badger JH, et al (2012) Structure, function and diversity of the healthy human microbiome. Nature 486: 207–214. 10.1038/nature11234 22699609 PMC3564958

[17] Douglas AE (2015) Multiorganismal Insects: Diversity and Function of Resident Microorganisms. Annual Review of Entomology 60: 17–34. 10.1146/annurev-ento-010814-020822 25341109 PMC4465791

[18] Berasategui A, Shukla S, Salem H, Kaltenpoth M (2016) Potential applications of insect symbionts in biotechnology. Applied Microbiology and Biotechnology 100: 1567–1577. 10.1007/s00253-015-7186-9 26659224 PMC4737797

[19] Lukasik P, van Asch M, Guo HF, Ferrari J, Godfray HCJ (2013) Unrelated facultative endosymbionts protect aphids against a fungal pathogen. Ecology Letters 16: 214–218. 10.1111/ele.12031 23137173

[20] Salem H, Bauer E, Strauss AS, Vogel H, Marz M, et al (2014) Vitamin supplementation by gut symbionts ensures metabolic homeostasis in an insect host. Proceedings of the Royal Society B-Biological Sciences 281: 20141838. 10.1098/rspb.2014.1838 PMC4213650 25339726

[21] Florez LV, Biedermann PHW, Engl T, Kaltenpoth M (2015) Defensive symbioses of animals with prokaryotic and eukaryotic microorganisms. Natural Product Reports 32: 904–936. 10.1039/c5np00010f 25891201

[22] Hamby KA, Becher PG (2016) Current knowledge of interactions between *Drosophila suzukii* and microbes, and their potential utility for pest management. Journal of Pest Science 89: 621–630.

[23] Sampson TR, Mazmanian SK (2015) Control of Brain Development, Function, and Behavior by the Microbiome. Cell Host & Microbe 17: 565–576. 25974299 10.1016/j.chom.2015.04.011 PMC4442490

[24] Klock MM, Barrett LG, Thrall PH, Harms KE (2015) Host promiscuity in symbiont associations can influence exotic legume establishment and colonization of novel ranges. Diversity and Distributions 21: 1193–1203.

[25] Taerum SJ, Konecny A, De Beer ZW, Cibrian-Tovar D, Wingfield MJ (2016) Population genetics and symbiont assemblages support opposing invasion scenarios for the red turpentine beetle (*Dendroctonus valens*). Biological Journal of the Linnean Society 118: 486–502.

[26] Hayward J, Horton TR, Pauchard A, Nunez MA (2015) A single ectomycorrhizal fungal species can enable a Pinus invasion. Ecology 96: 1438–1444. 26236856 10.1890/14-1100.1

[27] Uchitel A, Omacini M, Chaneton EJ (2011) Inherited fungal symbionts enhance establishment of an invasive annual grass across successional habitats. Oecologia 165: 465–475. 10.1007/s00442-010-1740-z 20686788

[28] Shapira M (2016) Gut Microbiotas and Host Evolution: Scaling Up Symbiosis. Trends in Ecology & Evolution 31: 539–549. 27039196 10.1016/j.tree.2016.03.006

[29] Brune A, Dietrich C (2015) The Gut Microbiota of Termites: Digesting the Diversity in the Light of Ecology and Evolution. Annual Review of Microbiology 69: 145–166. 10.1146/annurev-micro-092412-155715 26195303

[30] Rokhsefat S, Lin AF, Comelli EM (2016) Mucin-Microbiota Interaction During Postnatal Maturation of the Intestinal Ecosystem: Clinical Implications. Digestive Diseases and Sciences 61: 1473–1486. 10.1007/s10620-016-4032-6 26792279

[31] Holmes E, Li JV, Marchesi JR, Nicholson JK (2012) Gut Microbiota Composition and Activity in Relation to Host Metabolic Phenotype and Disease Risk. Cell Metabolism 16: 559–564. 10.1016/j.cmet.2012.10.007 23140640

[32] Rastmanesh R (2011) High polyphenol, low probiotic diet for weight loss because of intestinal microbiota interaction. Chemico-Biological Interactions 189: 1–8. 10.1016/j.cbi.2010.10.002 20955691

[33] Walter J, Ley R (2011) The Human Gut Microbiome: Ecology and Recent Evolutionary Changes. Annual Review of Microbiology 65: 411–429. 10.1146/annurev-micro-090110-102830 21682646

[34] Wetterer JK (2009) Worldwide spread of the ghost ant, *Tapinoma melanocephalum* (Hymenoptera: Formicidae). Myrmecological News 12: 23–33.

[35] Espadaler X, Espejo F (2002) *Tapinoma melanocephalum* (Fabricius, 1793), a new exotic ant in Spain (Hymenoptera, Fonnicidae). Orsis Organismes I Sistemes 17 101–104.

[36] Meer RKV, Jaffe K, Cedeno A (1990) Applied Myrmecology: a world perspective. Boulder: Westview Press Inc.

[37] Smith EH, Whitman RC (1992) Field guide to structural pests: National Pest Management Association, Dunn Loring, VA.

[38] Giraud T, Pedersen JS, Keller L (2002) Evolution of supercolonies: The Argentine ants of southern Europe. Proceedings of the National Academy of Sciences of the United States of America 99: 6075–6079. 10.1073/pnas.092694199 11959924 PMC122904

[39] Tsutsui ND, Case TJ (2001) Population genetics and colony structure of the argentine ant (*Linepithema humile*) in its native and introduced ranges. Evolution 55: 976–985. 11430657 10.1554/0014-3820(2001)055[0976:pgacso]2.0.co;2

[40] Thomas ML, Becker K, Abbott K, Feldhaar H (2010) Supercolony mosaics: two different invasions by the yellow crazy ant, *Anoplolepis gracilipes*, on Christmas Island, Indian Ocean. Biological Invasions 12: 677–687.

[41] Human KG, Gordon DM (1999) Behavioral interactions of the invasive Argentine ant with native ant species. Insectes Sociaux 46: 159–163.

[42] Human KG, Gordon DM (1996) Exploitation and interference competition between the invasive Argentine ant, *Linepithema humile*, and native ant species. Oecologia 105: 405–412. 10.1007/BF00328744 28307114

[43] Zeng L, Lu YY, He XF, Zhang WQ, Liang GW (2005) Identification of red imported fire ant, *Solenopsis invicta*, to invade mainland China and infestation in Wuchuan, Guangdong. Entomological Knowledge 02: 144–148.

[44] Lu YY, Wu BQ, Zeng L, Xu YJ (2012) Comparison of Foraging Ability Between *Solenopsis invicta* and *Tapinoma melanocephalum* (Hymenoptera: Formicidae). Sociobiology 59: 1015–1024.

[45] Zhou AM, Liang GW, Lu YY, Zeng L, Xu YJ (2014) Interspecific competition between the red imported fire ant, *Solenopsis invicta* Buren and ghost ant, *Tapinoma melanocephalum* Fabricius for honeydew resources produced by an invasive mealybug, *Phenacoccus solenopsis* Tinsiley. Arthropod-Plant Interactions 8: 469–474.

[46] McCutchan JH, Lewis WM, Kendall C, McGrath CC (2003) Variation in trophic shift for stable isotope ratios of carbon, nitrogen, and sulfur. Oikos 102: 378–390.

[47] BassiriRad H, Constable JVH, Lussenhop J, Kimball BA, Norby RJ, et al (2003) Widespread foliage delta N-15 depletion under elevated CO2: inferences for the nitrogen cycle. Global Change Biology 9: 1582–1590.

[48] Oberst S, Lai JCS, Evans TA (2016) Termites utilise clay to build structural supports and so increase foraging resources. Scientific Reports 6: 20990 10.1038/srep20990 26854187 PMC4745099

[49] Langille MGI, Zaneveld J, Caporaso JG, McDonald D, Knights D, et al (2013) Predictive functional profiling of microbial communities using 16S rRNA marker gene sequences. Nature Biotechnology 31: 814–821. 10.1038/nbt.2676 23975157 PMC3819121

[50] Hosokawa T, Koga R, Kikuchi Y, Meng XY, Fukatsu T (2010) *Wolbachia* as a bacteriocyte-associated nutritional mutualist. Proceedings of the National Academy of Sciences of the United States of America 107: 769–774. 10.1073/pnas.0911476107 20080750 PMC2818902

[51] Song Z, LU Y, Xu Y, Huang J, Liang G, et al (2010) Dynamic of native ants on the lawn with the invasion of *Solenopsis invicta* Buren. Acta Ecologica Sinica 30: 1287–1295.

[52] Rice ES, Silverman J (2013) Submissive behaviour and habituation facilitate entry into habitat occupied by an invasive ant. Animal Behaviour 86: 497–506.

[53] Kiesecker JM, Blaustein AR (1997) Population differences in responses of red-legged frogs (*Rana aurora*) to introduced bullfrogs. Ecology 78: 1752–1760.

[54] Lennartsson T, Tuomi J, Nilsson P (1997) Evidence for an evolutionary history of overcompensation in the grassland biennial *Gentianella campestris* (Gentianaceae). American Naturalist 149: 1147–1155. 10.1086/286043 18811268

[55] Trussell GC, Smith LD (2000) Induced defenses in response to an invading crab predator: An explanation of historical and geographic phenotypic change. Proceedings of the National Academy of Sciences of the United States of America 97: 2123–2127. 10.1073/pnas.040423397 10681425 PMC15764

[56] Phillips BL, Shine R (2004) Adapting to an invasive species: Toxic cane toads induce morphological change in Australian snakes. Proceedings of the National Academy of Sciences of the United States of America 101: 17150–17155. 10.1073/pnas.0406440101 15569943 PMC535375

[57] Mitchell MJ, Mills EL, Idrisi N, Michener R (1996) Stable isotopes of nitrogen and carbon in an aquatic food web recently invaded by *Dreissena polymorpha* (Pallas). Canadian Journal of Fisheries and Aquatic Sciences 53: 1445–1450.

[58] Shokal U, Yadav S, Atri J, Accetta J, Kenney E, et al (2016) Effects of co-occurring Wolbachia and Spiroplasma endosymbionts on the *Drosophila* immune response against insect pathogenic and non-pathogenic bacteria. BMC microbiology 16: 16 10.1186/s12866-016-0634-6 26862076 PMC4746768

[59] Brownlie JC, Cass BN, Riegler M, Witsenburg JJ, Iturbe-Ormaetxe I, et al (2009) Evidence for Metabolic Provisioning by a Common Invertebrate Endosymbiont, *Wolbachia pipientis*, during Periods of Nutritional Stress. Plos Pathogens 5: e1000368 10.1371/journal.ppat.1000368 19343208 PMC2657209

[60] Salvetti E, Fondi M, Fani R, Torriani S, Felis GE (2013) Evolution of lactic acid bacteria in the order Lactobacillales as depicted by analysis of glycolysis and pentose phosphate pathways. Systematic and Applied Microbiology 36: 291–305. 10.1016/j.syapm.2013.03.009 23735786

[61] Imhoff JF (2005) *Enterobacteriales*. Boston, MA: Springer US.

[62] Fraenkel DG, Vinopal RT (1973) Carbohydrate Metabolism in Bacteria. Microbiology 27: 69–100.

[63] Houadria MYI, Klimes P, Fayle TM, Gullan PJ (2018) Host-plant dissections reveal contrasting distributions of *Crematogaster* ants and their symbionts in two myrmecophytic *Macaranga* species. Ecological Entomology 43: 601–611.

[64] Lukasik P, Newton JA, Sanders JG, Hu Y, Moreau CS, et al (2017) The structured diversity of specialized gut symbionts of the New World army ants. Molecular Ecology 26: 3808–3825. 10.1111/mec.14140 28393425

[65] Maruyama M, Parker J (2017) Deep-Time Convergence in Rove Beetle Symbionts of Army Ants. Current Biology 27: 920–926. 10.1016/j.cub.2017.02.030 28285995

[66] Russell JA, Moreau CS, Goldman-Huertas B, Fujiwara M, Lohman DJ, et al (2009) Bacterial gut symbionts are tightly linked with the evolution of herbivory in ants. Proceedings of the National Academy of Sciences of the United States of America 106: 21236–21241. 10.1073/pnas.0907926106 19948964 PMC2785723

[67] Correa CC, Ballard JWO (2014) What can symbiont titres tell us about co-evolution of *Wolbachia* and their host? Journal of Invertebrate Pathology 118: 20–27. 10.1016/j.jip.2014.02.009 24594301

[68] Serbus LR, White PM, Silva JP, Rabe A, Teixeira L, et al (2015) The Impact of Host Diet on *Wolbachia* Titer in Drosophila. Plos Pathogens 11: e1004777 10.1371/journal.ppat.1004777 25826386 PMC4380406

[69] Ponton F, Wilson K, Holmes A, Raubenheimer D, Robinson KL, et al (2015) Macronutrients mediate the functional relationship between *Drosophila* and *Wolbachia*. Proceedings of the Royal Society B-Biological Sciences 282: 20142029. 10.1098/rspb.2014.2029 PMC4298205 25520356

[70] Kerkut GA (1985) Comprehensive insect physiology, biochemistry, and pharmacology: Pergamon Press.

[71] Puchta O, Wille H (1956) A parasitic bacterium in the mid-intestine of *Solenobia triquetrella*. Z Parasitenkd 17: 400–418. 13558449 10.1007/BF00267097

[72] Shigenobu S, Watanabe H, Hattori M, Sakaki Y, Ishikawa H (2000) Genome sequence of the endocellular bacterial symbiont of aphids *Buchnera sp* APS. Nature 407: 81–86. 10.1038/35024074 10993077

[73] Douglas AE (2009) The microbial dimension in insect nutritional ecology. Functional Ecology 23: 38–47.

[74] Hendry AP, Kinnison MT (2001) An introduction to microevolution: rate, pattern, process. Genetica 112: 1–8. 11838760

[75] Cheng Q, Jing QL, Spear RC, Marshall JM, Yang ZC, et al (2016) Climate and the Timing of Imported Cases as Determinants of the Dengue Outbreak in Guangzhou, 2014: Evidence from a Mathematical Model. Plos Neglected Tropical Diseases 10: e0004417 10.1371/journal.pntd.0004417 26863623 PMC4749339

[76] Zheng CY, Yang F, Zeng L, Vargo EL, Xu YJ (2018) Genetic diversity and colony structure of *Tapinoma melanocephalum* on the islands and mainland of South China. Ecology and Evolution 8: 5427–5440. 10.1002/ece3.4065 29938063 PMC6010919

[77] Wu BQ, Lu YY, Zeng L, Song ZD, Liang GW (2009) Foraging intensity of ants in *Solenopsis invicta* Buren (Hymenoptera: Formicidae) invaded and un-invaded habitats. Chinese Journal of Applied Ecology 20: 2513–2518. 20077713

[78] Carlin NF, Holldobler B (1986) The kin recognition system of carpenter ant (*Camponotus spp*).1.hierarchical cues in small colonies. Behavioral Ecology and Sociobiology 19: 123–134.

[79] Feldhaar H, Gebauer G, Bluthgen N (2010) Stable isotopes: past and future in exposing secrets of ant nutrition (Hymenoptera: Formicidae). Myrmecological News 13: 3–13.

[80] Cheng DF, Guo ZJ, Riegler M, Xi ZY, Liang GW, et al (2017) Gut symbiont enhances insecticide resistance in a significant pest, the oriental fruit fly *Bactrocera dorsalis* (Hendel). Microbiome 5: 13 10.1186/s40168-017-0236-z 28143582 PMC5286733

[81] Schloss PD, Westcott SL, Ryabin T, Hall JR, Hartmann M, et al (2009) Introducing mothur: Open-Source, Platform-Independent, Community-Supported Software for Describing and Comparing Microbial Communities. Applied and Environmental Microbiology 75: 7537–7541. 10.1128/AEM.01541-09 19801464 PMC2786419

[82] Huse SM, Welch DM, Morrison HG, Sogin ML (2010) Ironing out the wrinkles in the rare biosphere through improved OTU clustering. Environmental Microbiology 12: 1889–1898. 10.1111/j.1462-2920.2010.02193.x 20236171 PMC2909393

[83] Edgar RC (2013) UPARSE: highly accurate OTU sequences from microbial amplicon reads. Nature Methods 10: 996–998. 10.1038/nmeth.2604 23955772

[84] Segata N, Izard J, Waldron L, Gevers D, Miropolsky L, et al (2011) Metagenomic biomarker discovery and explanation. Genome Biology 12: R60 10.1186/gb-2011-12-6-r60 21702898 PMC3218848

[85] Zhou JZ, He ZL, Yang YF, Deng Y, Tringe SG, et al (2015) High-Throughput Metagenomic Technologies for Complex Microbial Community Analysis: Open and Closed Formats. Mbio 6: pii: e02288-02214. 10.1128/mBio.02288-14 PMC4324309 25626903

[86] Ortiz-Alvarez R, Fierer N, de los Rios A, Casamayor EO, Barberan A (2018) Consistent changes in the taxonomic structure and functional attributes of bacterial communities during primary succession. Isme Journal 12: 1658–1667. 10.1038/s41396-018-0076-2 29463893 PMC6018800

[87] Tamura K, Peterson D, Peterson N, Stecher G, Nei M, et al (2011) MEGA5: molecular evolutionary genetics analysis using maximum likelihood, evolutionary distance, and maximum parsimony methods. Mol Biol Evol 28: 2731–2739. 10.1093/molbev/msr121 21546353 PMC3203626

[88] Benjamini Y, Yekutieli D (2001) The control of the false discovery rate in multiple testing under dependency. The annals of statistics 29: 1165–1188.

