# Supplementary figures and images for: Symbiotic microbiota may reflect host adaptation by resident to invasive ant species
Source: PLoS Pathog. 2019 Jul 19;15(7):e1007942. doi: 10.1371/journal.ppat.1007942 (PMC6668852; doi:10.1371/journal.ppat.1007942)

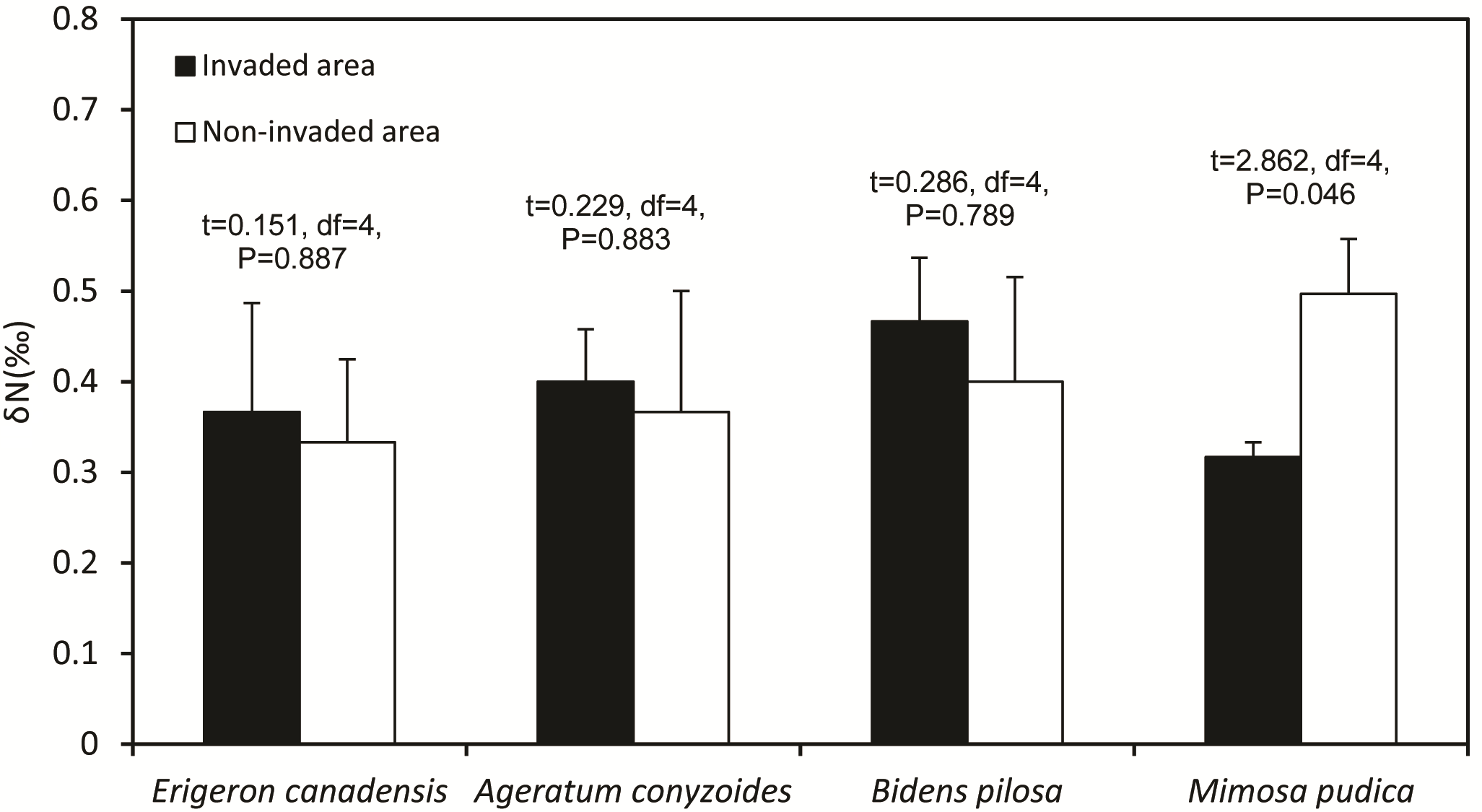

Supplement: S1 Fig — (TIF) [file ppat.1007942.s001.tif]

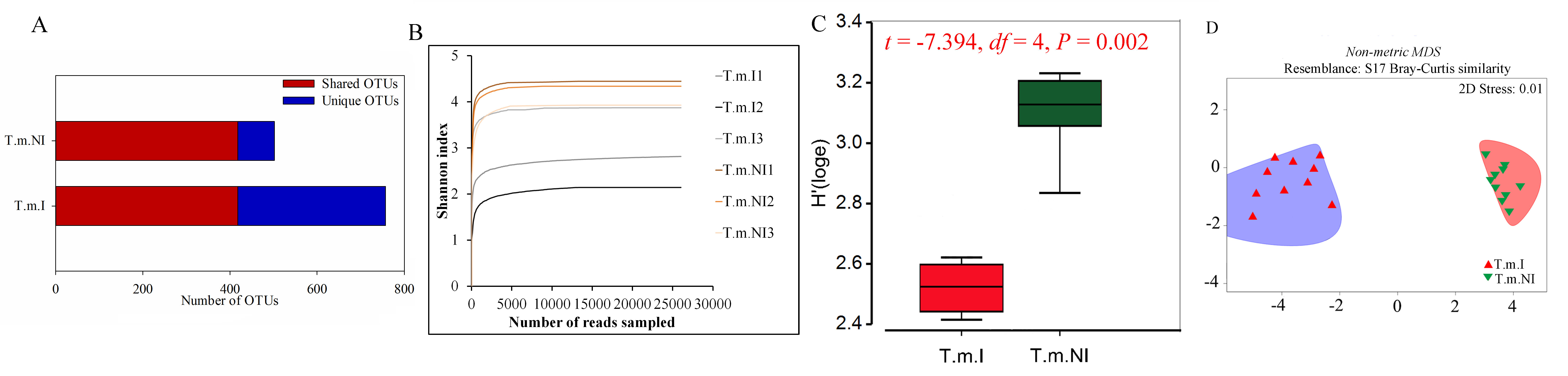

Supplement: S2 Fig — Community structure of bacterial symbionts from workers of T. melanocephalum in S. invicta invaded and non-invaded areas. Shared and unique OTUs in bacterial symbionts from workers of T. melanocephalum from the two areas (A); Rarefaction analysis based on the Shannon index for bacterial symbionts from workers. The index values are shown on the y-axis and the number or reads sampled are shown on the x-axis (B). Alpha diversity (Shannon index) (mean ± SE) of bacterial symbionts from workers of T. melanocephalum from the two areas (C); NMDS plot analyses based on Bray Curtis distances. T.m.I: T. melanocephalum in S. invicta invaded areas (D); T.m.NI: T. melanocephalum in S. invicta non-invaded areas. (TIF) [file ppat.1007942.s002.tif]

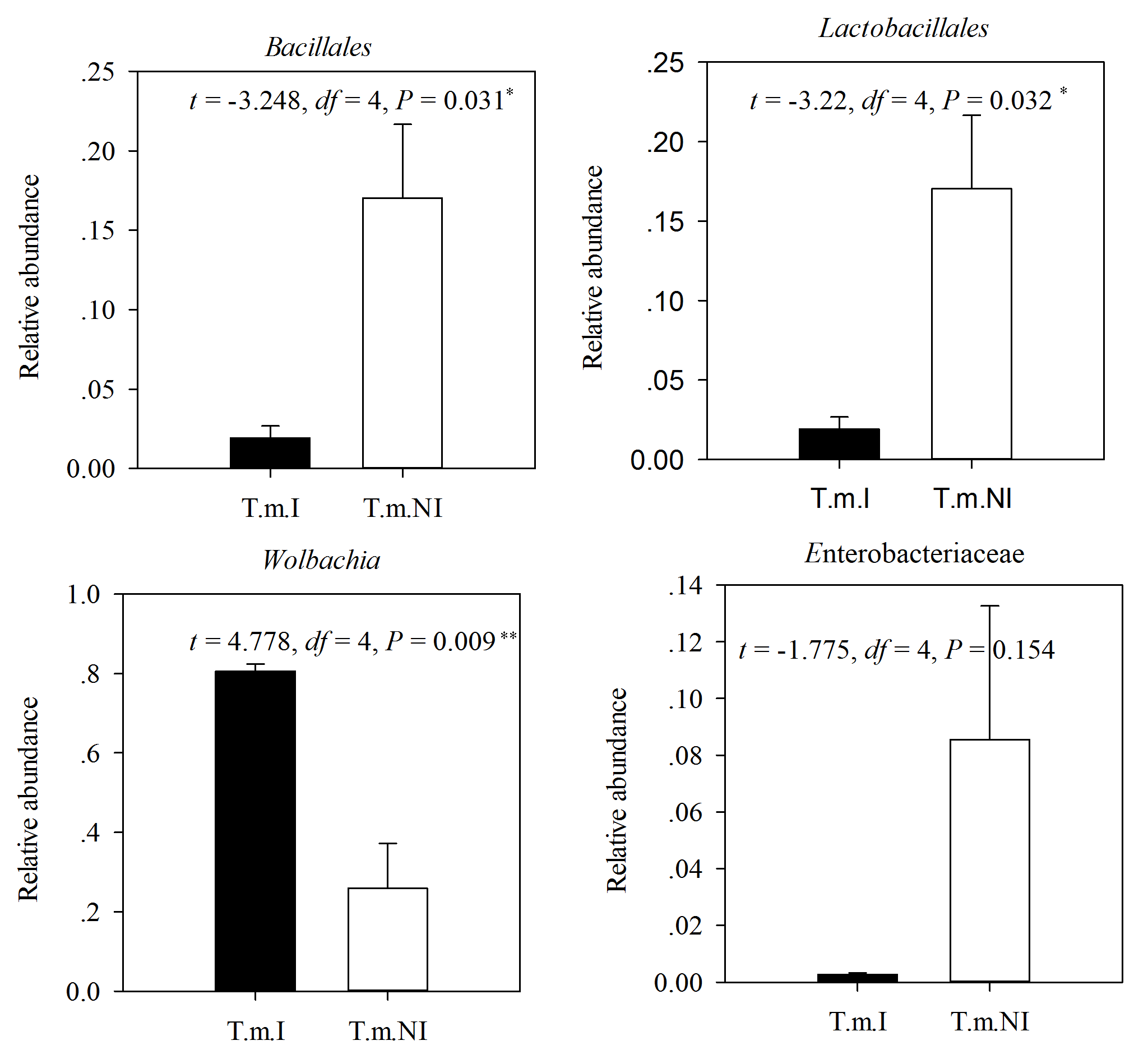

Supplement: S3 Fig — (TIF) [file ppat.1007942.s003.TIF]

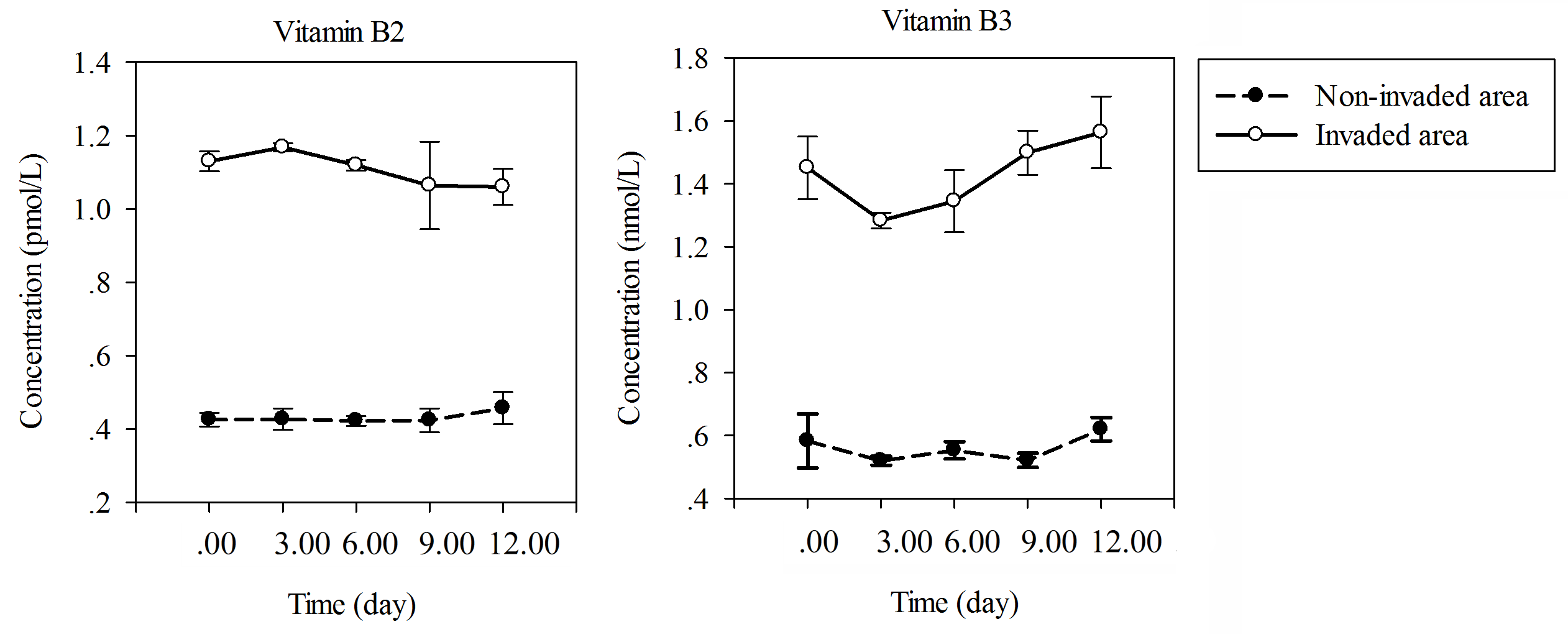

Supplement: S4 Fig — (TIF) [file ppat.1007942.s004.tif]

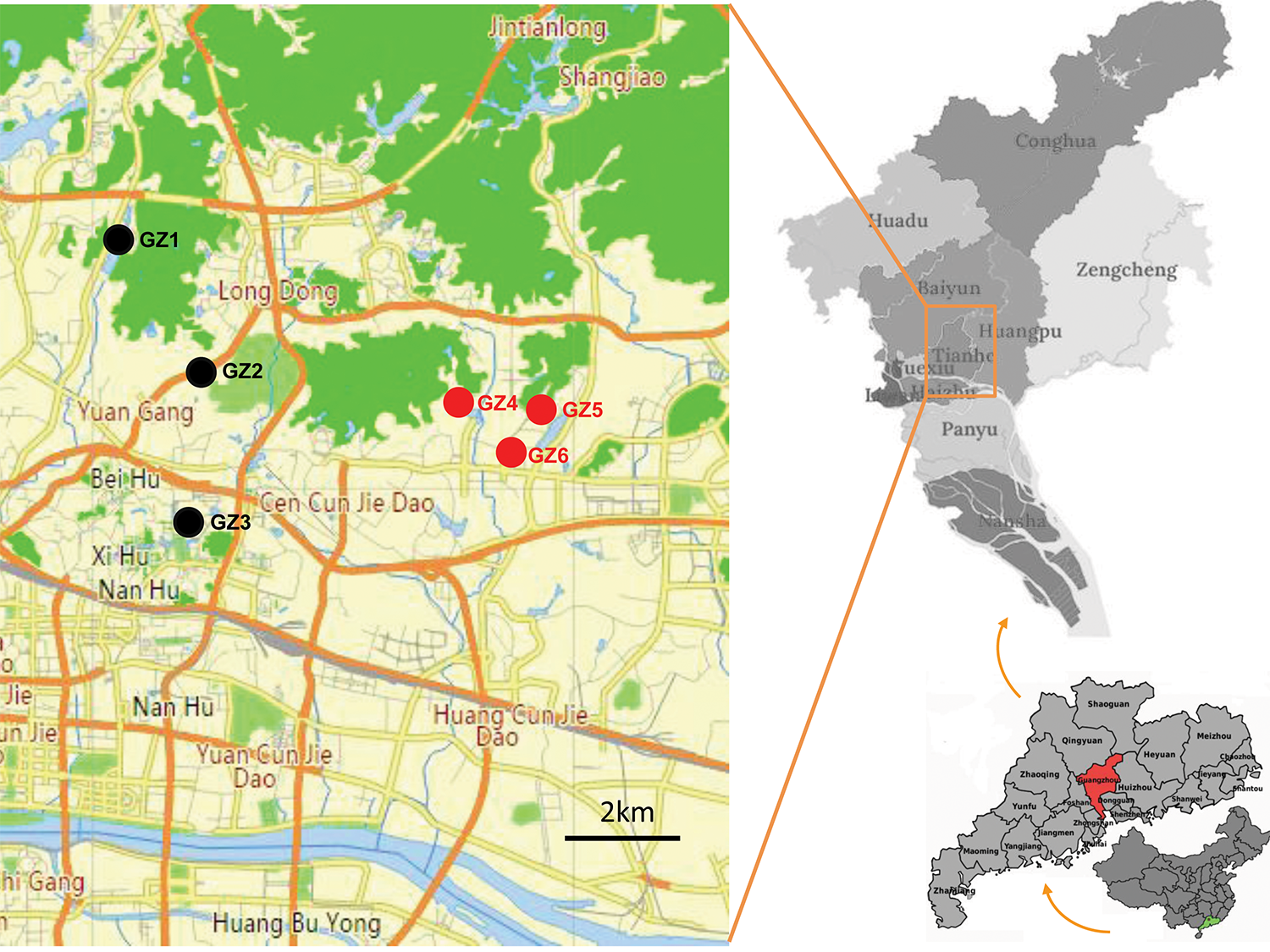

Supplement: S5 Fig — The map for study sites was adapted from the Wikipedia website (https://en.wikipedia.org/wiki/File:Guangzhou_city_map_plan_China_Level_12_English.svg, https://countrydigest.org/guangzhou-population/, https://upload.wikimedia.org/wikipedia/commons/3/31/Guangdong_administrative_divisions_2009_1level-fr.svg). (TIF) [file ppat.1007942.s005.tif]

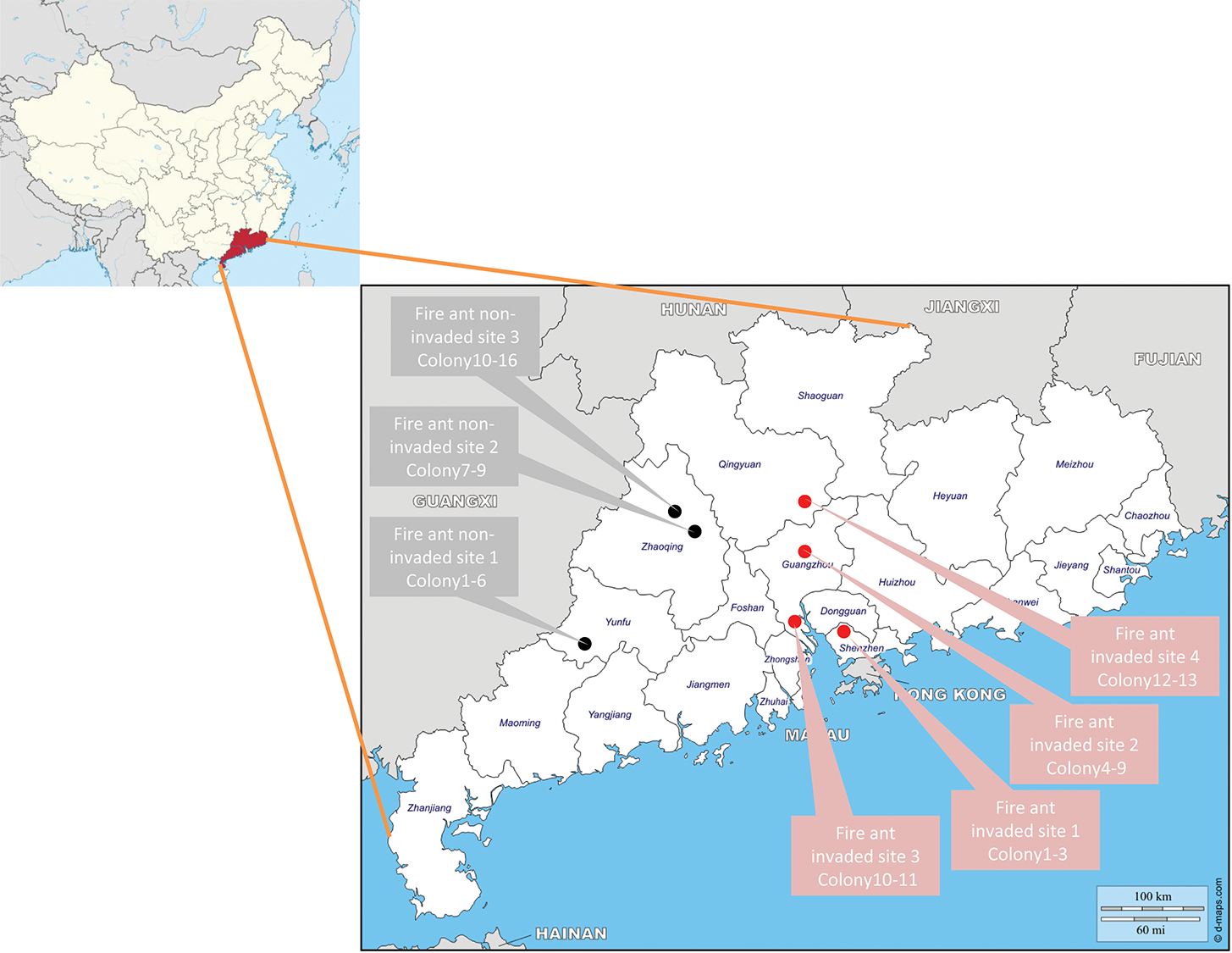

Supplement: S6 Fig — The map for study sites was adapted from the website (https://en.wikipedia.org/wiki/Guangdong, http://d-maps.com/m/asia/china/guangdong/guangdong19.gif). (TIF) [file ppat.1007942.s006.tif]

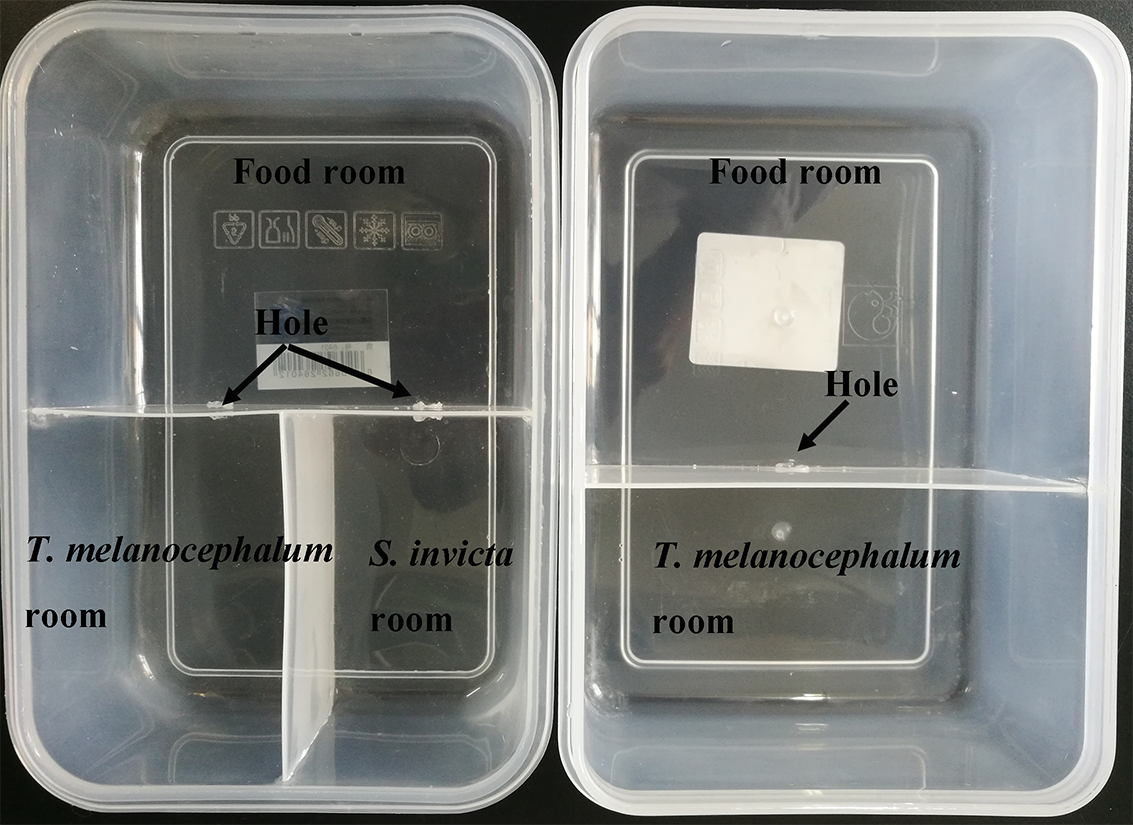

Supplement: S7 Fig — Left: treatment; Right: control. (TIF) [file ppat.1007942.s007.tif]
