# Supplementary material for: Symbiotic microbiota may reflect host adaptation by resident to invasive ant species
Source: PLoS Pathog. 2019 Jul 19;15(7):e1007942. doi: 10.1371/journal.ppat.1007942 (PMC6668852; doi:10.1371/journal.ppat.1007942)
Supplement: S1 Table — (DOCX) [file ppat.1007942.s008.docx]

Table S1 Number of ants per species captured in *S. invicta* invaded and non-invaded area

| Ant species | Invaded area | | | Non-invaded area | | |
| --- | --- | --- | --- | --- | --- | --- |
|  | Site 1 | Site 2 | Site 3 | Site 1 | Site 2 | Site 3 |
| *Monomorium subopacum* | 0 | 0 | 0 | 47 | 63 | 34 |
| *Technomyrmex albipes* | 12 | 5 | 98 | 0 | 0 | 0 |
| *Crematogaster biroi* | 0 | 0 | 0 | 2 | 73 | 45 |
| *Paratrechina bourbonica* | 41 | 6 | 6 | 20 | 18 | 7 |
| *Monomorium pharaonic* | 584 | 242 | 210 | 4 | 72 | 71 |
| *Crematogaster rogenhoferi* | 0 | 0 | 0 | 7 | 6 | 6 |
| *Tapinoma melanocephalum* | 1646 | 1261 | 1127 | 142 | 269 | 251 |
| *Odontoponera transversa* | 5 | 0 | 0 | 10 | 4 | 11 |
| *Solenopsis invicta* | 2399 | 2832 | 2774 | 0 | 0 | 0 |
| *Leptogenys chinensis* | 0 | 0 | 0 | 2 | 0 | 1 |
| *Camponotus albosparsus* | 0 | 0 | 0 | 10 | 7 | 11 |
| *Paratrechina flavipes* | 54 | 43 | 63 | 60 | 110 | 77 |
| *Pheidole noda* | 0 | 0 | 0 | 170 | 205 | 170 |
| *Pheidole fervida* | 572 | 950 | 955 | 384 | 234 | 227 |
| *Plagiolepis Rothneyi* | 2 | 0 | 90 | 20 | 4 | 22 |
| *Cardiocondyla nuda* | 3 | 0 | 1 | 0 | 0 | 0 |
| *Camponotus nicobaresis* | 0 | 0 | 0 | 7 | 4 | 0 |
| *Pheidole pieli* | 0 | 0 | 0 | 100 | 224 | 85 |
| *Tetramorium caespitum* | 0 | 0 | 0 | 0 | 2 | 0 |
| *Pheidologeton diversus* | 3 | 7 | 11 | 14 | 50 | 2 |
| *Tetramorium smithi* | 0 | 0 | 0 | 2 | 30 | 8 |
| *Polyrhachis dives* | 0 | 2 | 1 | 0 | 0 | 0 |
| *Tetramorium bicarinatum* | 34 | 4 | 46 | 82 | 32 | 31 |
| *Monamorium* *concolor* | 0 | 0 | 0 | 25 | 42 | 37 |
| *Pheidole yeensis* | 126 | 157 | 6 | 7 | 106 | 88 |
| *Paratrechina longicornis* | 30 | 0 | 9 | 20 | 42 | 31 |
